# Antibacterial and Antioxidant Activities of Isolated Compounds from *Prosopis africana* Leaves

**DOI:** 10.1155/2022/4205823

**Published:** 2022-02-17

**Authors:** Lambert Yanda, Simplice J. N. Tatsimo, Jean-De-Dieu Tamokou, Germaine Matsuete-Takongmo, Sylvie Carolle Meffo-Dongmo, Alain Meli Lannang, Norbert Sewald

**Affiliations:** ^1^Department of Chemistry, Faculty of Sciences, The University of Maroua, Maroua, Cameroon; ^2^Natural Product and Environmental Chemistry Group (NAPEC), Department of Chemistry, Higher Teachers' Training College, University of Maroua, Box 55, Maroua, Cameroon; ^3^Research Unit of Microbiology and Antimicrobial Substances, Faculty of Sciences, University of Dschang, Dschang, P. O Box 67, Cameroon; ^4^Department of Chemical Engineering, School of Chemical Engineering and Mineral Industries, University of Ngaoundere, Ngaoundere, Cameroon; ^5^Department of Chemistry, Organic and Bioorganic Chemistry, Bielefeld University, Bielefeld, Germany

## Abstract

*Prosopis africana* (G. &Perr.) Taub (Mimosaceae) is a large tree native to dry tropical Africa and characteristic of dry leguminous forests. Different parts of this plant are used to treat wounds, skin infection, and to fight against cancer. Literature review indicated various pharmacological properties. Despite these medicinal properties, the chemical composition studies remain limited. This study aims to isolate and characterize secondary metabolites from *P. africana* leaves and evaluate their antibacterial and antioxidant properties. Air-dried powdered leaves of *P. africana* were macerated in methanol at room temperature and partitioned with ethyl acetate. The EtOAc extract was subjected successively to flash and column chromatographies in order to isolate compounds. The structure of the isolates was determined with help of spectroscopic data including 1D and 2D NMR experiments and comparison with literature data. The antibacterial activities were evaluated via determination of minimum inhibitory concentration (MIC) and minimum bactericidal concentration (MBC). The antioxidant activities were evaluated via gallic acid equivalent antioxidant capacity (GEAC) and diphenyl-1-picrylhydrazyl (DPPH) free radical scavenging assays. The chemical investigation of the EtOAc extract led to the isolation of seven compounds: (2*E*, 6*E*) farnesylamine (1), myricetin-3-O-rhamnoside (2), bis(2-ethylhexyl) benzene-1,2-dicarboxylate (3), lupeol (4), *ß*-sitosterol (5), stigmasterol glycoside (6), and a mixture of bis(2-ethylhexyl) benzene-1,2-dicarboxylate (3) and bis(2-ethylhexyl) benzene-1,4-dicarboxylate (7) in ratio 1 : 2. Compound 1 is described here for the first time as a natural product with complete ^1^H and ^13^C assignments. Compounds 3 and 7 were identified as artefacts from dichloromethane. Sesquiterpene amine (1) is reported in *Prosopis* genus for the first time. Antibacterial and antioxidant activities of isolated compounds were investigated. Among the tested samples, the EtOAc extract and compound 2 exhibited the highest antioxidant (EC_50_ = 5.67–77.56 *μ*g/mL; GEAC = 36.58–89.28 *μ*g/mL) and antibacterial (MIC = 8–64 *μ*g/mL) activities against gram-negative and gram-positive bacteria. The EtOAc extract and compound 2 from *P. africana* exhibited antibacterial activity through bacteriolytic effects and reduction of the antioxidant defenses in the bacterial cells. Furthermore, the chemotaxonomic significance of isolated compounds was discussed. The antibacterial and antioxidant activities of ethyl acetate extract and compound 2 can justify the traditional uses of *P. africana* leaves for the treatment of diseases related to bacterial infections. The presence of compounds 1, 2, and 4 in this plant should also be considered as valuable chemotaxonomic features.

## 1. Introduction

Most cardinal infectious diseases which account for more than 85% of the mortality from infection worldwide such as tuberculosis, AIDS, malaria, diarrheal diseases, and acute respiratory infections have serious problems in the treatment due to widespread emergence of antimicrobial resistance [1]. Antibacterial resistance results in increased morbidity and mortality from treatment failures and increased healthcare costs. Medicinal plants have different groups of active metabolites and are used traditionally against infectious diseases, so they could serve as complementary medicine to treat bacterial infections.

Biochemical pathways or cellular mechanisms produce free reactive oxygen species (ROS) as an end product [2] which are harmful to living cells and can cause mutation, cardiovascular disease, and Alzheimer's disease [3]. Oxidative stress is due to an imbalance between the production of ROS and a biological system's ability to readily detoxify the reactive intermediates or easily repair the resulting damage. Usually, synthetic antioxidants are used to attenuate the destructive effects of free radicals. However, they have shown to be toxic on human health and the development of naturally occurring antioxidants from plant origin could be fruitful to humankind [4].

*Prosopis africana* (G. &Perr.) Taub (Mimosaceae), commonly called Africa mesquite and “Wa” in Tupuri tribe in Chad, is a large tree native to dry tropical Africa and characteristic of dry leguminous forests. It is widely distributed in the Sahelian, Sudanese, and Guinean savannas. The tree is 10–20 m high, can reach 0.4–0.8 m in diameter, and has light green foliage [5]. It is one of the 44 species of *Prosopis* genus wide found in south of Chad. Almost all parts of this tree are used in traditional medicine. In Chad, decoction of the trunk bark of *P. africana* is used to heal wounds and to fight against cancer. Leaves are used to treat headache and toothache as well as various other ailments. Leaves and barks are combined to treat rheumatism, remedies for skin diseases, caries, and fevers. The roots are diuretic and are used to treat gonorrhea, toothache, stomachache, dysentery, and bronchitis [6]. Previous studies mainly on the chemical investigation of *P. africana* species reveal the presence of some isolated compounds including three flavones [7, 8], two steroids [8], one triterpene [9], three fatty alcohols [8], and seven alkaloids [10]. In this study, we report the isolation of seven known compounds (1–7) including one new natural sesquiterpene derivative (1), a flavone glycoside (2), two phthalate derivatives (3, 7), two steroids (5, 6), and one triterpene (4). We also report the antibacterial and antioxidant activities and the chemotaxonomic significance of isolated compounds.

## 2. Materials and Methods

### 2.1. General Experimental Procedures

The ^1^H, ^13^C, and 2D NMR spectra were recorded on a Bruker AMX-500 spectrometer.

Homonuclear^1^H–^1^H connectivities were determined by COSY-45 experiment. One-bond ^1^H–^13^C connectivities were determined by HMQC, while two- and three-bond ^1^H–^13^C connectivities were determined by HMBC. ^1^H and ^13^C chemical shifts are reported in *δ* (ppm) with reference to TMS. Coupling constants (J) were measured in Hz. The EIMS were recorded on a double-focusing mass spectrometer (Varian MAT 311A). Column chromatography was carried out on silica gel 60 (0.060–0.200 mm, 70–230 mesh, Alfa Aesar) and with Sephadex LH-20 (GE Healthcare Europe GmbH). Precoated TLC sheets (POLYGRAM^®^ SIL G/UV_254_) were used to check the purity of compounds, and detection was accomplished by visualizing with a UV lamp at 254 and 365 nm, followed by spraying with a solution of 10% sulfuric acid in distilled water (v/v) and then heating.

### 2.2. Plant Material

*Prosopis africana* leaves were collected on 24 November 2018 in Kocporaï area, East Mayo-Kebbi region, Chad. It was identified by Prof. Tchobsala, botanist in the Department of Biology, Faculty of Science, University of Maroua, Cameroon. The identification was confirmed at the National Herbarium, Yaoundé, Cameroon, by comparison with a specimen of *P. africana* with voucher no. 7016 SRF/Cam.

### 2.3. Extraction and Isolation

Air-dried powdered leaves of *P. africana* (4 kg) were macerated in 12 liters of methanol (3 × 72 hours) at room temperature. The combined methanol extract was concentrated using a rotary evaporator (70°C) to yield 700 g crude extract. 500 g of the current extract was then subjected to liquid-liquid fractionation with ethyl acetate (EtOAc) to obtain 190 g ethyl acetate extract. The EtOAc extract (150 g) was subjected to a silica gel flash column chromatography (FC) eluted with normal hexane-CH_2_Cl_2_ (1 : 0, 9 : 1, 8 : 2, 7 : 3, 3 : 2, 1 : 1, 4 : 4, and 3 : 7) and CH_2_Cl_2_-MeOH (1 : 0, 19 : 1, 8 : 2, 1 : 1, 2 : 8, and 0 : 1) to afford 113 fractions. These fractions were combined into five main fractions, F1 (2.04 g), F2 (15.30 g), F3 (10.70 g), F4 (20.07 g), and F5 (40.70 g), based on their TLC profile. These fractions were subjected to further purification using silica gel to afford 7 compounds. Fraction F1 (2.04 g) eluted with normal hexane-EtOAc (1 : 0, 49 : 1, 19 : 1, and 8 : 2) yielded 1 (618 mg) and fraction F3 (15.30 g) eluted with the same system yielded 6 (706 mg) and 5 (141 mg). Fraction F4 (20.07 g) eluted with normal hexane-EtOAc (19 : 1, 4 : 1, 7 : 3, 3 : 2, 1 : 1, and 0 : 1) and CH_2_Cl_2_-MeOH (19 : 1, 9 : 1, 17 : 1, 4 : 1, 7 : 3, 1 : 1, and 0 : 1) gave 4 subfractions (F1'–F4'). Further purification of subfraction F1' (10.05 g**)** with normal hexane-EtOAc (46 : 1, 24 : 1, 47 : 3, 23 : 2, and 9 : 1) yielded 3 (2.63 g) and 6 (278 mg). Fraction F5 (40.70 g) eluted with CH_2_Cl_2_-MeOH (9 : 1, 17 : 1, 4 : 1, 15 : 1, and 7 : 3) gave 2 (38 mg) and 7 (156 mg). The identification of the structures of these compounds was done on the basis of 1D and 2D NMR experiments and by comparing with those described in the literature. As 3 and 7 were identified as plasticizers, solvents used were distilled, and these two compounds were found to be artefacts from CH_2_Cl_2_.

### 2.4. Spectroscopic Data of Compound 1

*(2E, 6E) Farnesylamine* (1): colorless oil. MS (ESI+): *m/z* 465.4 [2M + Na]^+^ (calcd for C_30_H_54_N_2_Na *m/z* 465.4). ^1^H NMR (500 MHz, CDCl_3_): 5.11–5.20 (3H, m, H-2, H-6, and H-10), 2.11 (4H, m, H-5 and H-9), 2.05 (2H, m, H-1), 2.01 (4H, m, H-4 and H-8), 1.71 (3H, s, H-12), and 1.63 (9H, s, H-13, H-14, and H-15). ^13^C NMR (125 MHz, CDCl_3_): 135.03 (C, C-3), 134.82 (C, C-7), 131.14 (C, C-11), 124.43 (CH, C-10), 124.33 (CH, C-2), 124.29 (CH, C-6), 39.77 (CH_2_, C-4), 39.75(CH_2_, C-8), 28.29 (CH_2_, C-1), 26.79 (CH_2_, C-5), 26.67 (CH_2_, C-9), 25.68 (CH_3_, C-12), 17.65 (CH_3_, C-13), 16.02 (CH_3_, C-14), and 15.98 (CH_3_, C-15).

### 2.5. Bioassays

#### 2.5.1. Antibacterial Activity

*(1) Bacteria and Growth Conditions*. The studied microorganisms consisted of two gram-positive (*Staphylococcus aureus* ATCC 25923 and *Enterococcus faecalis* ATCC 29212) and three gram-negative (*Pseudomonas aeruginosa* ATCC 76110, *Escherichia coli* ATCC 25922, and *Klebsiella pneumonia* 22) bacteria taken from the Research Unit of Microbiology and Antimicrobial Substances, Department of Biochemistry, University of Dschang, Cameroon. The bacterial species were grown at 37°C and maintained on nutrient agar (NA, Conda, Madrid, Spain).

*(2) Determination of Minimum Inhibitory Concentration (MIC) and Minimum Bactericidal Concentration (MBC)*. INT colorimetric assay [11] was used to determine the minimal inhibitory concentrations (MICs) of extracts/compounds against gram-negative and gram-positive bacteria. In brief, after subcultivation of bacterial species, a defined inoculum (1.5 × 106 CFU/mL) was added to the broth containing defined concentrations of the test samples as a two-fold dilution series or the controls and incubated at 35°C for 24 h. After this period, the MIC values of extracts/compounds were determined by adding 40 *μ*L of a 0.2 mg/mL p-iodonitrotetrazolium violet solution followed by a new incubation at 35°C for 30 min. Viable bacteria reduced the colorless dye to pink. MIC represented the lowest concentration that prevented this change and displayed full inhibition of bacterial growth. Ciprofloxacin (Sigma-Aldrich, Steinheim, Germany) was tested as reference antibacterial. All assays were made in independent replicates. For the determination of MBC values, a portion of broth (5 *μ*L) from each well that exhibited no growth of bacteria was inoculated on the surface of Mueller–Hinton agar plates and incubated at 35°C for 24 h. The lowest concentrations of test samples which gave no growth of bacteria after this subcultivation were taken as the MBC values.

*(3) Antibacterial Mechanism Studies*. The bacteriolytic, salt tolerance, and antioxidant enzyme assays were used to determine the mode of antibacterial action.

  Bacteriolytic assay: the time-kill kinetic method [12, 13] was used to determine the bacteriolytic activity of the EtOAc extract and compound 2 (the most active samples) against *Escherichia coli* ATCC 25922 and *Enterococcus faecalis* ATCC 29212. The starting inoculum was prepared by diluting full growth of bacterium in MHB and incubated at 37°C to produce an OD_600_ of 0.8. The samples were mixed with the starting bacterial suspension to a final concentration of 2 × MIC. The mixture was incubated for 0, 15, 30, 60, 120, and 240 min at 37°C under agitation at 150 rpm. After the incubation period, the optical density was read at 600 nm using a BIOBASE UV-Vis spectrophotometer. Blanks were made with the corresponding dilutions of test samples. Oxacillin and tubes without extract/compound were used as positive and negative controls, respectively. All the measurements were carried out in triplicate.
  Antioxidant enzyme activities: *Escherichia coli* ATCC 25922 and *Enterococcus faecalis* ATCC 29212 (1.5 × 10^8^ CFU/mL, 500 *μ*L) cultures from the late exponential growth phase were treated with MIC and ½ x MIC of EtOAc extract (500 *μ*L), compound 2 (500 *μ*L), and ciprofloxacin (500 *μ*L) solutions and incubated at 37°C for 24 h [13]. After centrifugation of the suspension at 3000 rpm for 5 min, the pellet was washed twice with PBS and resuspended in 500 *μ*L of cell lysate buffer (1 mM EDTA, 10 mM Tris-HCl, 0.1% Triton-X-100, and 150 mM NaCl) and finally incubated at 37°C for 1 h. Contents were then centrifuged at 3000 rpm for 5 min, and the supernatant was separated and used for catalase and superoxide dismutase (SOD) activity assays.
  Catalase activity: the catalase activity was evaluated by using a kit (Sigma, catalogue no. CAT100) with cell lysate. 25 *μ*L of 50 mM H_2_O_2_ was mixed with 750 *μ*L of assay buffer (50 mM) and 10 *μ*L of cell lysate. The content was incubated for 5 min. The reaction was arrested by the addition of 900 *μ*L of 15 mM sodium azide, and the content was thoroughly mixed. Then, 10 *μ*L of reaction mixture was mixed with 1 mL of color reagent (2 mM 3,5-dichloro-2-hydroxybenzenesulfonic acid and 0.25 mM 4-aminoantipyrine) and incubated for 15 min. The optical density was read at 520 nm, and the catalase activity was calculated by using the following equation [13]: [Δ*μ* moles (H_2_O_2_) × *d* × 100)/*V* × *t*], where Δ*μ* moles (H_2_O_2_) = difference in amount of H_2_O_2_ added to the reaction mixture between blank and given sample, *d* = dilution of the original sample for catalase reaction, *V* = sample volume in catalase reaction, and *t* = reaction duration (min).
  Superoxide dismutase (SOD) activity: the SOD activity was assessed using a kit (Sigma, catalogue no. 19160) in the cell lysate. The working solution of water-soluble tetrazolium salt (WST, 200 *μ*L) and enzyme solution (20 *μ*L) were mixed with 20 *μ*L of cell lysate. The content was incubated in the dark at 37°C for 20 min, and the optical density was monitored at 450 nm on a BioTek Synergy 2 multiplate reader. The SOD activity was calculated by using the following formula [13]: [(A_Blank1_ – A_Blank3_) – (A_Sample_ – A_Blank2_)/(A_Blank1_ – A_Blank3_) × 100], where Blank 1 contains ultrapure water, WST solution, and enzyme solution; Blank 2 contains sample solution, WST solution, and dilution buffer; and Blank 3 contains ultrapure water, WST solution, and dilution buffer.

#### 2.5.2. Antioxidant Assay

*(1) Gallic Acid Equivalent Antioxidant Capacity (GEAC) Assay*. The GEAC test was done as previously described [13, 14]. In brief, 20 *μ*L of purified laccase (1 mM stock solution) [15], 20 *μ*L of EtOAc extract/compound 2 at different concentrations, 10 *μ*L of ABTS (2,2′-azino-bis(3-ethylbenzothiazoline-6-sulfonic acid) (74 mM stock solution), and 950 *μ*L of acetate buffer (pH = 5.0, 100 mM) were mixed in a quartz cuvette. After 240 s reaction time, the content of the generated ABTS^**●+**^ radical was monitored at 420 nm and converted to gallic acid equivalents by the use of a calibration curve (Pearson's correlation coefficient: *r* = 0.999) constructed with 0, 4, 10, 14, 28, 56, and 84 *μ*M gallic acid standards rather than Trolox. Experiments were performed in independent replicates.

*(2) Diphenyl-1-picrylhydrazyl (DPPH) Free Radical Scavenging Assay*. The DPPH assay with the extracts and compounds was performed as previously described [16]. EC_50_ (*μ*g/mL) represents the amount of extract/compound necessary to inhibit by 50% the optical density of free radical DPPH [16]. Vitamin C was used as a reference antioxidant. All the assays were done in independent replicates.

## 3. Results and Discussion

### 3.1. Structure Elucidation of Isolated Compounds

**Figure 1:**
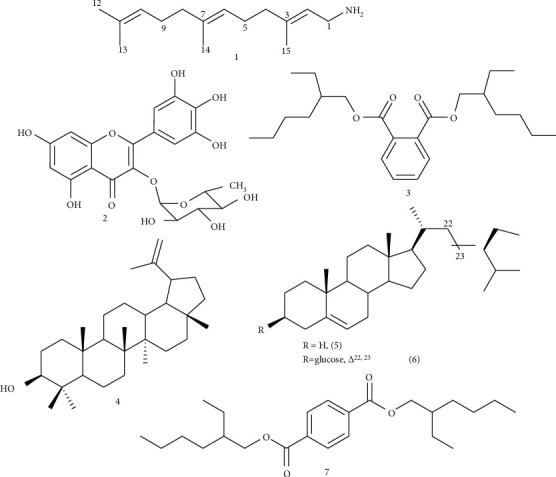
Structure of isolated compounds (1–7).

Phytochemical investigation of the ethyl acetate extract of *P. africana* leaves yielded to the isolation of seven compounds named (2*E*, 6*E*) farnesylamine (1) [17], myricetin-3-O-rhamnoside (2) [17–21], bis(2-ethylhexyl) benzene-1,2-dicarboxylate (3) [22–24], lupeol (4) [25], *ß*-sitosterol (5) [8], stigmasterol glycoside (6), and a mixture of bis(2-ethylhexyl) benzene-1,2-dicarboxylate (3) and bis(2-ethylhexyl) benzene-1,4-dicarboxylate (**7**) [22] in ratio 1 : 2 (Figure 1). Compounds 3 and 7 were identified as artefacts from nondistilled dichloromethane. Compound 1 was isolated and described here as a natural product for the first time.

Compound 1 was obtained as colorless oil in hexane. It was assigned the formula C_15_H_27_N by the combination of the ESI-MS (+) (*m/z* 465.4, [2M + Na]^+^) and ^1^H and ^13^C-NMR data. The ^1^H NMR spectrum (Figure S2) showed six signals including a broad signal centered at *δ*_H_ 5.17 integrating for three olefinic protons and three multiplets at *δ*_H_ 2.01 (4H), 2.05 (2H), and 2.11 (4H) characteristic of five methylene groups. In the upfield area, two singlets at *δ*_H_ 1.63 (9H) and *δ*_H_ 1.71 (3H) characteristic of four deshielded methyl groups of aliphatic sesquiterpene skeleton [26] were observed. The ^13^C NMR spectrum (Figure S3), in association with DEPT-135 spectrum (Figure S4), showed the presence of fifteen carbon atoms including four methyls (*δ*_c_ 15.98–25.68), five methylenes (*δ*_C_ 26.67–39.77), three methines (*δ*_C_ 124.29–124.43), and three quaternary carbons (*δ*_C_ 131.14–135.03), which confirmed the sesquiterpene skeleton for compound 1 [27, 28]. Analyses of HMBC spectrum (Figure S7) indicated specific long-range correlation between the methyl protons at *δ*_H_ 1.63 and the carbons at *δ*_C_ 135.03, 134.82, 131.14, 124.29–124.43, 39.77, 39.75, and 25.68 and between the olefinic protons and carbons at *δ*_C_ 39.77, 39.75, 28.29, 26.79, 26.67, 17.65, 16.02, and 15.98. Further analyses of HMBC correlations confirmed the farnesyl skeleton for compound 1, and it was identified as farnesylamine [26, 27]. The configuration of the double bonds at positions 2 and 6 was established as *E* due to the resonance carbons at 39.77 (C-4) and 39.75 (C-8) in comparison with data published by Lamnaouer et al. [28] and Coppola and Prashad [27]. Therefore, the structure of compound 1 was deduced as (2*E*, 6*E*) farnesylamine, different to (2*Z*, 6*Z*) farnesylamine isolated by Kiplimo et al. [26] from the leaves of *Vernonia auriculifera*. Compound 1 was synthetized by Coppola and Prashad [27], and it is reported here for the first time as a natural product.

### 3.2. Biological Activities

#### 3.2.1. Antibacterial Activity

The MeOH and EtOAc extracts exhibited antibacterial activities against both gram-positive and gram-negative bacteria at varying degrees (Table 1). Compared to the MeOH extract, the smallest MIC values were obtained with the EtOAc extract, indicating that the fractionation improved the activity of the latter. The MeOH and EtOAc extracts displayed MBC values against all the tested bacterial species. Of all the compounds isolated, compound 2 was the most active, followed in decreasing order by the mixture 3 + 7, compounds 3, 6, and 4. However, compound 1 was found inactive on all the tested bacterial species. In comparison to the extracts and isolated compounds, the smallest MIC values (i.e., corresponding to the greatest antibacterial activities) were recorded with ciprofloxacin. The degree of sensitivity of the bacterial species and hence antibacterial activity of test samples is of the order *Enterococcus faecalis* ATCC 29212 > *Staphylococcus aureus* ATCC 25923 > *Escherichia coli* ATCC 25922 > *Pseudomonas aeruginosa* ATCC 76110 > *Klebsiella pneumoniae* 22, as shown by the minimum inhibitory concentrations. Since the tested bacteria are both gram-positive and gram-negative, it is likely that the mechanism of activity observed might not be linked with cell wall formation or disruption/interference with cell wall integrity. This suggests that the antibacterial activity may be linked to the inhibition of the molecular processes vital for the survival of the bacteria or the inhibition of key metabolic enzymes in the tested bacteria. The antibacterial activity of the *P. africana* extracts is in agreement with that of the previous reports [29–32]. However, to the best of our knowledge, no work has been reported on the antibacterial activity of the compounds isolated from *P. africana*. The MBC values of the MeOH and EtOAc extracts as well as compounds 2, 3, and the mixture 3 + 7 were equal to or 2 times greater than those of the MICs on the corresponding bacteria. This finding suggests that these samples have bactericidal effects (MBC/MIC ≤4) with respect to sensitive bacteria [16]. In contrast, compounds 4 and 6 did not exhibit MBC values against sensitive bacterial species. Hence, compounds 4 and 6 have bacteriostatic effects (MBC/MIC >4) on sensitive bacteria [16].

**Figure 2:**
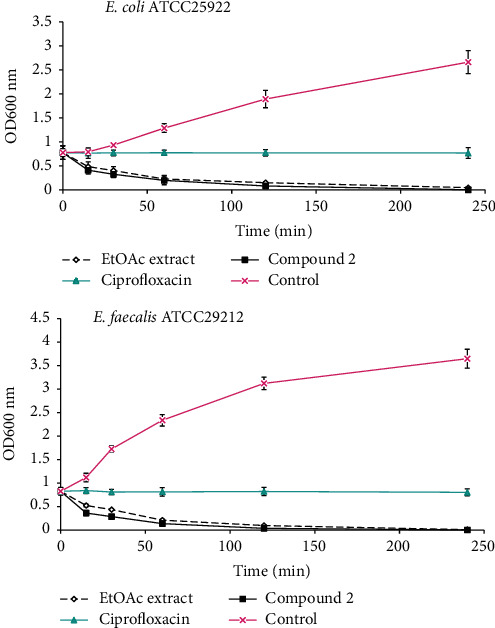
Bacteriolytic effect of EtOAc extract and compound 2 against *E. coli* and *E. faecalis.* The results represent the mean ± SD of the triplicate OD at each incubation time.

**Figure 3:**
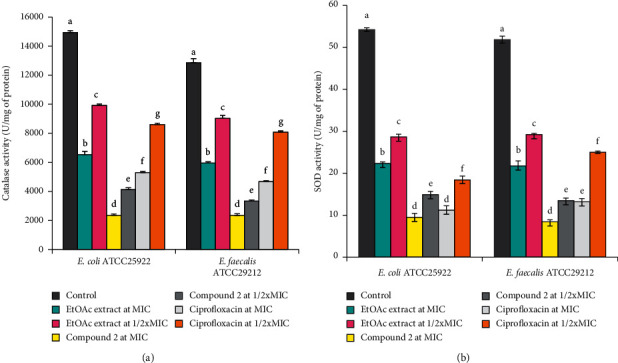
Antioxidant catalase (a) and superoxide dismutase (b) activities in *E. coli* and *E. faecalis* treated with EtOAc extract and compound 2. Bars represent the mean ± SD of three independent experiments carried out in triplicate. For the same bacteria and enzyme, values earmarked by different superscript letters (a–f) are significantly different according to one-way ANOVA and Waller-Duncan test; *p* < 0.05.

Mechanism of antibacterial activity:

1. Bacteriolytic activity: the bacteriolytic activity of the EtOAc extract and compound 2, which exhibited the highest antibacterial activity, was evaluated against *Escherichia coli* ATCC 25922 and *Enterococcus faecalis* ATCC 29212 using the time-kill kinetic method (Figure 2). It emerges from the results obtained that the EtOAc extract and compound 2 at the concentration of 2 x MIC showed significant decreases in optical densities in *E. coli* and *E. faecalis* suspensions as a function of time (Figure 2). These observations indicate the lysis of the tested bacterial cells in the presence of the EtOAc extract and compound 2 at the concentration of 2 x MIC. The effect of compound 2 was stronger than that of the EtOAc extract. In contrast, ciprofloxacin did not cause a decrease in optical density in *E. coli* and *E. faecalis* suspensions over time. Increases in optical densities were observed in *E. coli* and *E. faecalis* suspensions not treated with the extract/compound (i.e., control), suggesting the bacteria growth in the absence of the EtOAc extract and compound 2 over time.
2. Antioxidant enzyme activities: superoxide dismutase (SOD) catalyzes the dismutation of superoxide into hydrogen peroxide and is the first line of defense in bacterial cells against reactive oxygen species [13, 33], whereas catalase is involved in the detoxification of H_2_O_2_ by converting it into H_2_O and O_2_ with the help of heme cofactor [13, 34]. In the present study, antioxidant activity of the EtOAc extract and compound 2 was evaluated against bacterial catalase and superoxide dismutase at concentrations of ½ x MIC and 1 x MIC (Figure 3). Compared to the control (containing the bacterial culture not treated with the extract/compound), the EtOAc extract and compound 2 caused concentration-dependent significant inhibition (*p* < 0.05) on the catalase and superoxide dismutase activities independently of the tested bacteria (Figure 3). Moreover, whatever the concentration, the inhibitory effects of compound 2 against the two bacterial enzymes were generally greater than those of the EtOAc extract and ciprofloxacin, used as a reference antibiotic. These findings suggest that suppression of the SOD activity results in decreased conversion of O_2_^•−^ to H_2_O_2_ and consequently culminating in increased O_2_^•−^ levels and leads to oxidative stress-mediated toxicity against *E. coli* and *E. faecalis* [13]. Also, a decrease of catalase level caused by EtOAc extract/compound 2 might result in increased H_2_O_2_ level and leads to oxidative stress-mediated toxicity against the tested bacteria [13]. Altogether, the EtOAc extract and compound 2 displayed antibacterial activity through bacteriolytic effect and reduction of the antioxidant defenses in the bacterial cells. This is the first report on the mechanisms of antibacterial action of EtOAc extract and compound 2 from *P. africana* against pathogenic bacteria.

#### 3.2.2. Antioxidant Activity

The antioxidant activity was evaluated by determining the DPPH free radical scavenging activity as well as gallic acid equivalent antioxidant capacity (GEAC) of the extracts and compound 2 (Table 2). It emerges from the results obtained that, among the tested extracts, the EtOAc extract recorded the smallest EC_50_ value (i.e., corresponding to the greatest DPPH free radical scavenging activity) compared to the MeOH extract (Table 2). In contrast, the MeOH extract recorded the greatest gallic acid equivalent antioxidant capacity (i.e., corresponding to the greatest gallic acid equivalent antioxidant capacity) compared to the EtOAc extract. The DPPH free radical scavenging activity and the gallic acid equivalent antioxidant capacity of compound 2 were greater than those of the two extracts, suggesting that this compound is partially responsible for the antioxidant activities found in these plant extracts. The fact that the antioxidant activity of the extracts depends on the method used demonstrates that the test extracts contain several antioxidant compounds with several chemical mechanisms. In addition to compound 2, phenolic derivatives have been previously isolated from *P. africana* [7,8]. Phenolic compounds are known to scavenge active oxygen species and free radicals such as singlet oxygen, superoxide anion, and hydroxyl radicals [35].

### 3.3. Chemotaxonomic Significance

Previous phytochemical studies revealed that species of *Prosopis* genus are rich in piperidine alkaloids as well as several flavonoid glycosides [10, 36–38]. In this study, one sesquiterpene amine (1), one flavone glycoside (2), phthalate derivatives (artefacts) (3 and 7), one triterpene (4), and two steroids (5, 6) were isolated from *P. africana* leaves. To our knowledge, compounds (1, 2, and 4) are reported herein for the first time from *Prosopis* genus. Compounds 5 and 6 are already reported in *Prosopis* genus. (2*Z*, 6*Z*)-Farnesylamine has been isolated from *Vernonia auriculifera* for the first time [26] and was detected in an extract of *Monomorium fieldi* Forel from Australia [39]. It is the first time the isomer (2*E*, 6*E*)-farnesylamine is isolated from natural source, but has been previously synthetized by Coppola and Prashad [27]. Myricetin-3-O-rhamnoside (2) has been previously isolated from many other plants including *Liquidambar styraciflua* [20], *Searsia chirindensis L*. [40], and *Cercis chinensis* [14]. This study is the first report of myricetin-3-O-rhamnoside (2) from *Prosopis* genus.

## 4. Conclusion

The results of this study enrich the knowledge on phytochemistry of the plant and provide further information in regard to the possible chemotaxonomic markers in this species, the *Prosopis* genus as well as Mimosaceae family. It is noteworthy that compounds (1, 2, and 4) have not been reported from the *Prosopis* genus and therefore suggest new findings on chemotaxonomic information of the genus and additional constituents for the chemical diversity of *P. africana*. In addition, the good antibacterial and antioxidant activities of ethyl acetate extract and compound 2 are an asset to valorize the traditional uses of *P. africana* leaves for the treatment of diseases related to bacterial infections.

## Figures and Tables

**Table 1:** Antibacterial activity (MIC and MBC in *μ*g/mL) of extracts and isolated compounds against pathogenic bacteria.

Extracts/compounds	Inhibition parameters	*Staphylococcus aureus* ATCC 25923	*Enterococcus faecalis* ATCC 29212	*Pseudomonas aeruginosa* ATCC 76110	*Escherichia coli* ATCC 25922	*Klebsiella pneumoniae* 22
*MeOH extract*	MIC	32	64	64	128	32
	MBC	64	64	128	256	64
	MBC/MIC	2	1	2	2	2

*EtOAc extract*	MIC	64	32	64	64	64
	MBC	64	64	64	64	64
	MBC/MIC	1	2	1	1	1

1	MIC	>256	>256	>256	>256	>256
	MBC	nd	nd	nd	nd	nd
	MBC/MIC	nd	nd	nd	nd	nd

2	MIC	8	8	16	16	32
	MBC	16	8	32	16	32
	MBC/MIC	2	1	2	1	1

3	MIC	32	64	64	64	32
	MBC	64	64	128	128	64
	MBC/MIC	2	1	2	2	2

4	MIC	>256	128	128	256	>256
	MBC	nd	>256	>256	>256	nd
	MBC/MIC	nd	nd	nd	nd	nd

6	MIC	>256	128	128	128	>256
	MBC	nd	>256	>256	>256	nd
	MBC/MIC	nd	nd	nd	nd	nd

3 **+** 7	MIC	32	64	32	32	32
	MBC	64	64	64	64	64
	MBC/MIC	2	1	2	2	2

*Ciprofloxacin*	MIC	4	2	4	8	2
	MBC	8	4	8	8	4
	MBC/MIC	2	2	2	1	2

MIC, minimum inhibitory concentrations; MBC, minimum bactericidal concentrations; nd, not determined.

**Table 2:** Antioxidant activities (EC_50_ and GEAC in *μ*g/mL) of extracts and compound 2 from *Prosopis africana*.

Extracts/compounds	DPPH free radical scavenging activity (EC_50_)	Gallic acid equivalent antioxidant capacity (GEAC)
MeOH extract	93.66 ± 0.94^a^	43.41 ± 0.79^a^
EtOAc extract	77.56 ± 1.62^b^	36.58 ± 84^b^
2	5.67 ± 0.73^c^	89.28 ± 0.74^c^
Vitamin C	1.29 ± 0.33^d^	—

EC_50_: equivalent concentrations of test samples scavenging 50% of DPPH radical. Data represent the mean ± SD of three independent experiments carried out in triplicate. In the same column, values earmarked by different superscript letters (a–d) are significantly different according to one-way ANOVA and Waller-Duncan test; *p* < 0.05.

## Data Availability

The datasets generated and analysed during the current study are available from the corresponding author upon reasonable request.

## Abbreviations

^13^C-NMR: Carbon-13 nuclear magnetic resonance
^1^H-NMR: Proton nuclear magnetic resonance
2D NMR: Two-dimensional nuclear magnetic resonance
ATCC: American Type Culture Collection
CC: Column chromatography
COSY: Correlated spectroscopy
DMSO: Dimethyl sulfoxide
DPPH: Diphenyl-1-picrylhydrazyl
EtOAc: Ethyl acetate
GEAC: Gallic acid equivalent antioxidant capacity
HMBC: Heteronuclear multiple bond connectivities
HSQC: Heteronuclear single quantum coherence
IR: Infrared
MBC: Minimum bactericidal concentration
MDR: Multidrug resistant
MeOH: Methanol
MHA: Mueller–Hinton agar
MHB: Mueller–Hinton broth
MIC: Minimum inhibitory concentration
MBC: Minimum bactericidal concentration
NA: Nutrient agar
*n*-BuOH: *n*-Butanol
NMR: Nuclear magnetic resonance
Rf: Retention factor
TLC: Thin-layer chromatography
TMS: Tetramethylsilane
UV: Ultraviolet.

## Supplementary Materials

Supplementary Materials Figure S1: mass spectra ESI (+) of compound 1. Figure S2: ^1^H NMR spectrum (500 MHz, CDCl_3_) of compound 1. Figure S3:^13^C NMR spectrum (125 MHz, CDCl_3_) of compound 1. Figure S3a: ^13^C NMR spectrum (125 MHz, CDCl_3_) of compound 1. Figure S3b: ^13^C NMR spectrum (125 MHz, CDCl_3_) of compound 1. Figure S4: DEPT-135 NMR spectrum (125 MHz, CDCl_3_) of compound 1. Figure S5: COSY-45 spectrum of compound 1. Figure S6: HMQC spectrum of compound 1. Figure S7: HMBC spectrum of compound 1. FIGURE S8: mass spectrum ESI of compound 2. Figure S9: ^1^H NMR spectrum (500 MHz, CD_3_OD) of compound 2. Figure S10: ^13^C NMR spectrum (125 MHz, CD_3_OD) of compound 2. Figure S11: DEPT-135 spectrum (125 MHz, CD_3_OD) of compound 2. Figure S12: COSY-45 spectrum of compound 2. Figure S13: HMQC spectrum of compound 2. Figure S14: HMBC spectrum of compound 2. FIGURE S15: mass spectrum ESI of compound 3. Figure S16: ^1^H NMR spectrum (500 MHz, CDCl_3_) of compound 3. Figure S17: ^13^C NMR spectrum (125 MHz, CDCl_3_) of compound 3. Figure S18: DEPT-135 NMR spectrum (125 MHz, CDCl_3_) of compound 3. Figure S19: COSY-45 spectrum of compound 3. Figure S20: HMQC spectrum of compound 3. Figure S21: HMBC spectrum of compound 3. Figure S22: ^1^H NMR spectrum (500 MHz, CDCl_3_) of compound 5. Figure S23: mass spectrum ESI (+) of compound 7. Figure S24: ^1^H NMR (500 MHz, CDCl_3_) of compound 7. Figure S25: ^13^C NMR (125 MHz, CDCl_3_) of compound 7. Figure S25a: ^13^C NMR (125 MHz, CDCl_3_) of compound 7. Figure S25b: ^13^C NMR (125 MHz, CDCl_3_) of compound 7. Figure S26: ^13^C NMR (125 MHz, CDCl_3_) of compound **7**. Figure S26a: ^13^C NMR (125 MHz, CDCl3) of compound 7. Figure S27: COSY-45 spectrum of compound 7. Figure S28: HMQC spectrum of compound 7. Figure S29: HMBC spectrum of compound 7.
