# Supplementary material for: Antibacterial and Antioxidant Activities of Isolated Compounds from Prosopis africana Leaves
Source: Int J Anal Chem. 2022 Feb 17;2022:4205823. doi: 10.1155/2022/4205823 (PMC8872693; doi:10.1155/2022/4205823)
Supplement: Supplementary Materials — Figure S1: mass spectra ESI (+) of compound 1. Figure S2: 1H NMR spectrum (500 MHz, CDCl3) of compound 1. Figure S3:13C NMR spectrum (125 MHz, CDCl3) of compound 1. Figure S3a: 13C NMR spectrum (125 MHz, CDCl3) of compound 1. Figure S3b: 13C NMR spectrum (125 MHz, CDCl3) of compound 1. Figure S4: DEPT-135 NMR spectrum (125 MHz, CDCl3) of compound 1. Figure S5: COSY-45 spectrum of compound 1. Figure S6: HMQC spectrum of compound 1. Figure S7: HMBC spectrum of compound 1. FIGURE S8: mass spectrum ESI of compound 2. Figure S9: 1H NMR spectrum (500 MHz, CD3OD) of compound 2. Figure S10: 13C NMR spectrum (125 MHz, CD3OD) of compound 2. Figure S11: DEPT-135 spectrum (125 MHz, CD3OD) of compound 2. Figure S12: COSY-45 spectrum of compound 2. Figure S13: HMQC spectrum of compound 2. Figure S14: HMBC spectrum of compound 2. FIGURE S15: mass spectrum ESI of compound 3. Figure S16: 1H NMR spectrum (500 MHz, CDCl3) of compound 3. Figure S17: 13C NMR spectrum (125 MHz, CDCl3) of compound 3. Figure S18: DEPT-135 NMR spectrum (125 MHz, CDCl3) of compound 3. Figure S19: COSY-45 spectrum of compound 3. Figure S20: HMQC spectrum of compound 3. Figure S21: HMBC spectrum of compound 3. Figure S22: 1H NMR spectrum (500 MHz, CDCl3) of compound 5. Figure S23: mass spectrum ESI (+) of compound 7. Figure S24: 1H NMR (500 MHz, CDCl3) of compound 7. Figure S25: 13C NMR (125 MHz, CDCl3) of compound 7. Figure S25a: 13C NMR (125 MHz, CDCl3) of compound 7. Figure S25b: 13C NMR (125 MHz, CDCl3) of compound 7. Figure S26: 13C NMR (125 MHz, CDCl3) of compound 7. Figure S26a: 13C NMR (125 MHz, CDCl3) of compound 7. Figure S27: COSY-45 spectrum of compound 7. Figure S28: HMQC spectrum of compound 7. Figure S29: HMBC spectrum of compound 7. [file 4205823.f1.pdf]

Antibacterial and antioxidant activities of isolated compounds from *Prosopis africana* leaves

Lambert Yanda[1,2], Simplice J.N. Tatsimo[2,*], Jean-De-Dieu Tamokou[3,**], Germaine Matsuete-Takongmo[3], Sylvie Carolle Meffo-Dongmo[3], Alain Meli Lannang[2,4], Norbert Sewald[5]

[1]*Department of Chemistry, Faculty of Sciences, the University of Maroua, Maroua, Cameroon*
[2]*Natural Product and Environmental Chemistry group (NAPEC), Department of Chemistry, Higher Teachers' Training College, University of Maroua, Box 55 Maroua, Cameroon*
[3]*Research Unit of Microbiology and Antimicrobial Substances, Faculty of Sciences, University of Dschang, Dschang, P. O Box 67, Cameroon.*
[4]*Department of Chemical Engineering, School of Chemical Engineering and Mineral Industries, University of Ngaoundere, Ngaoundere, Cameroon*
[5]*Department of Chemistry, Organic and Bioorganic Chemistry, Bielefeld University, Bielefeld, Germany*

Correspondance: *Professor Simplice J.N. Tatsimo, Natural Product and Environmental Chemistry group (NAPEC), Department of Chemistry, Higher Teachers' Training College, University of Maroua, P.O. Box. 55 Maroua-Cameroon, Tel.: +237 677355476, Email: jtatsimo@yahoo.com

**Professor Jean-de-Dieu Tamokou, Research Unit of Microbiology and Antimicrobial Substances, Department of Biochemistry, Faculty of Science, University of Dschang, P.O. Box 67 Dschang, Cameroon, Tel: +237 677 000 897. E-mail: jtamokou@yahoo.fr / jean.tamokou@univ-dschang.org

FIGURE captions

FIGURE S1: Mass spectra ESI (+) of compounds **1**. 4

FIGURE S2: ^{1}H NMR spectrum (500 MHz, $CDCl_3$) of compound **1**. 4

FIGURE S3:^{13}C NMR spectrum (125 MHz, $CDCl_3$) of compound **1**. 5

FIGURE S3a: ^{13}C NMR spectrum (125 MHz, $CDCl_3$) of compound **1**. 5

FIGURE S3b: ^{13}C NMR spectrum (125 MHz, $CDCl_3$) of compound **1**. 6

FIGURE S4: DEPT-135 NMR spectrum (125 MHz, $CDCl_3$) of compound **1**. 6

FIGURE S5: COSY-45 spectrum of compound **1**. 7

FIGURE S6: HMQC spectrum of compound **1**. 7

FIGURE S7: HMBC spectra of compound **1**. 8

FIGURE S8: Mass spectrum ESI of compounds **2**. 8

FIGURE S9: ^{1}H NMR spectrum (500 MHz, CD_3OD) of compound **2**. 9

FIGURE S10: ^{13}C NMR spectrum (125 MHz, CD_3OD) of compound **2**. 9

FIGURE S11: DEPT-135 spectrum (125 MHz, CD_3OD) of compound **2**. 10

FIGURE S12**:** COSY-45 spectrum of compound **2**. 10

FIGURE S13**:** HMQC spectrum of compound **2**. 11

FIGURE S14: HMBC spectrum of compound **2**. 12

FIGURE S15: Mass spectrum ESI of compound **3**. 12

FIGURE S16: ^{1}H NMR spectrum (500 MHz, $CDCl_3$) of compound **3**. 13

FIGURE S17: ^{13}C NMR spectrum (125 MHz, $CDCl_3$) of compound **3**. 13

FIGURE S18: DEPT-135 NMR spectrum (125 MHz, $CDCl_3$) of compound **3**. 14

FIGURE S19: COSY-45 spectrum of compound **3**. 14

FIGURE S20**:** HMQC spectrum of compound **3**. 15

FIGURE S21: HMBC spectrum of compound **3**. 15

FIGURE S22: ^{1}H NMR spectrum (500 MHz, $CDCl_3$) of compound **5**. 16

FIGURE S23: Mass spectrum ESI (+) of compound **7**. 16

FIGURE S24: ^{1}H NMR (500 MHz, $CDCl_3$) of compound **7**. 17

FIGURE S25: ^{13}C NMR (125 MHz, $CDCl_3$) of compound **7**. 17

FIGURE S25a: ^{13}C NMR (125 MHz, $CDCl_3$) of compound **7**. 18

FIGURE S25b: ^{13}C NMR (125 MHz, $CDCl_3$) of compound **7**. 18

FIGURE S26:^{13}C NMR (125 MHz, $CDCl_3$) of compound **7**. 19

FIGURE S26a:13C NMR (125 MHz, $CDCl_3$) of compound **7**. 19

FIGURE S27: COSY-45 spectrum of compound **7**. 20

FIGURE S28: HMQC spectrum of compound **7**. 20

FIGURE S29: HMBC spectrum of compound **7** 21

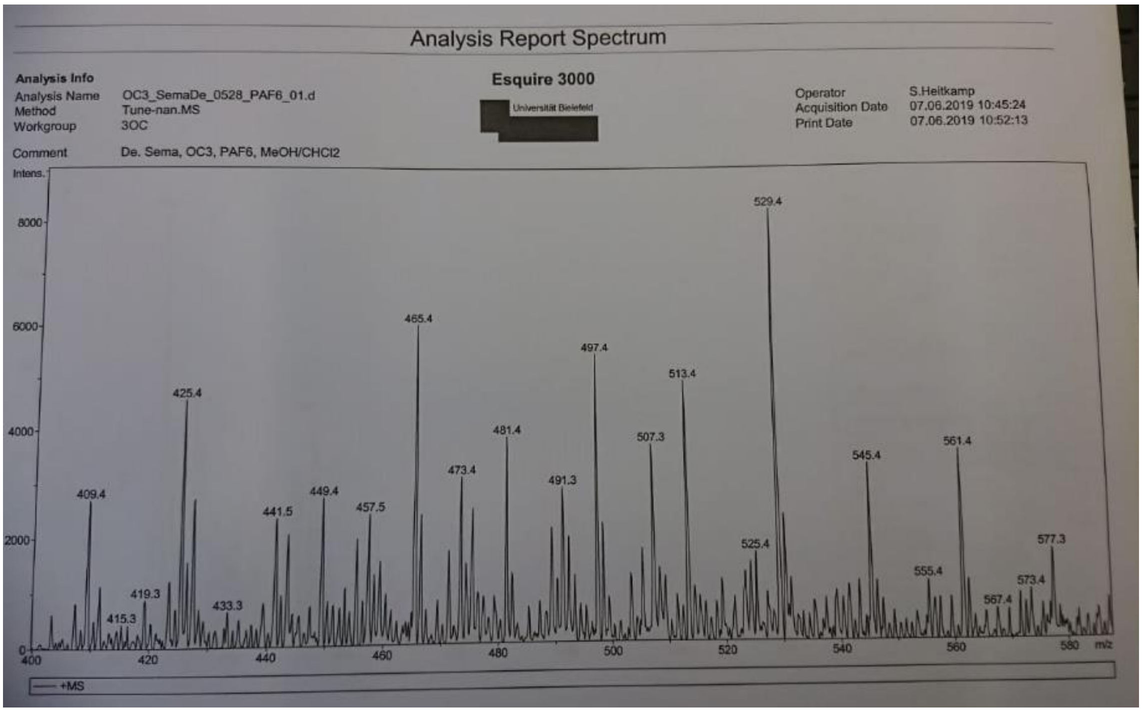

FIGURE S1: Mass spectra ESI (+) of compounds **1**

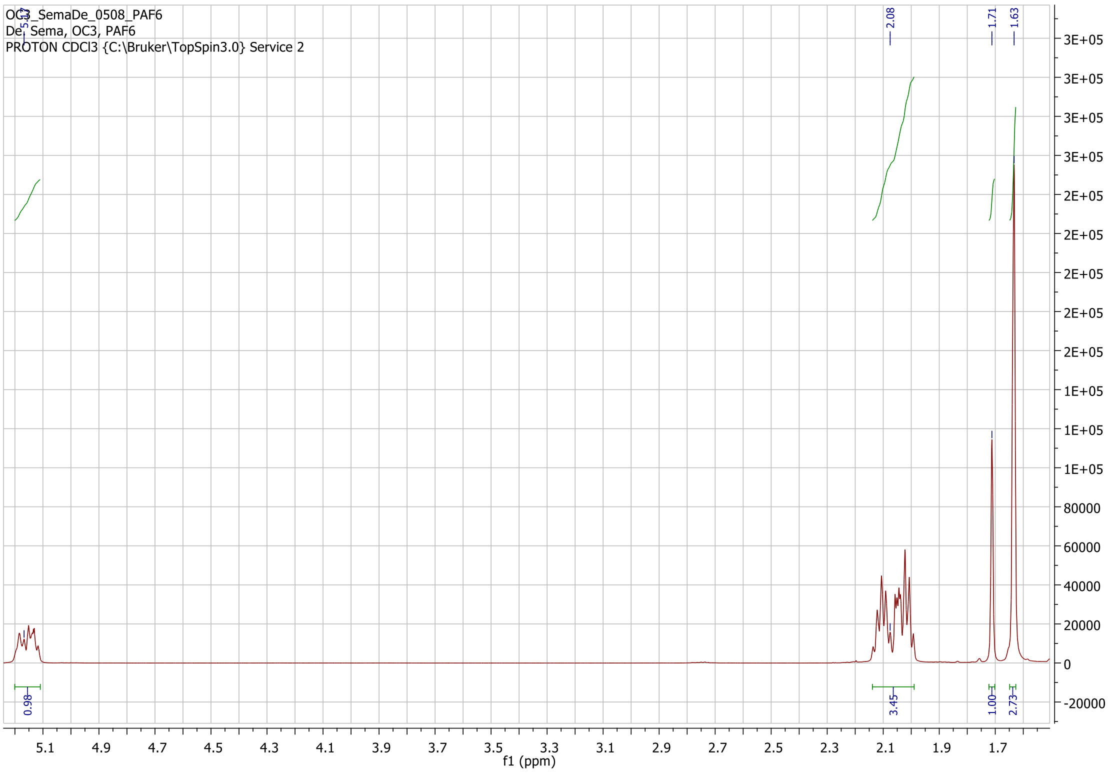

FIGURE S2: ^{1}H NMR spectrum (500 MHz, $CDCl_3$) of compound **1**

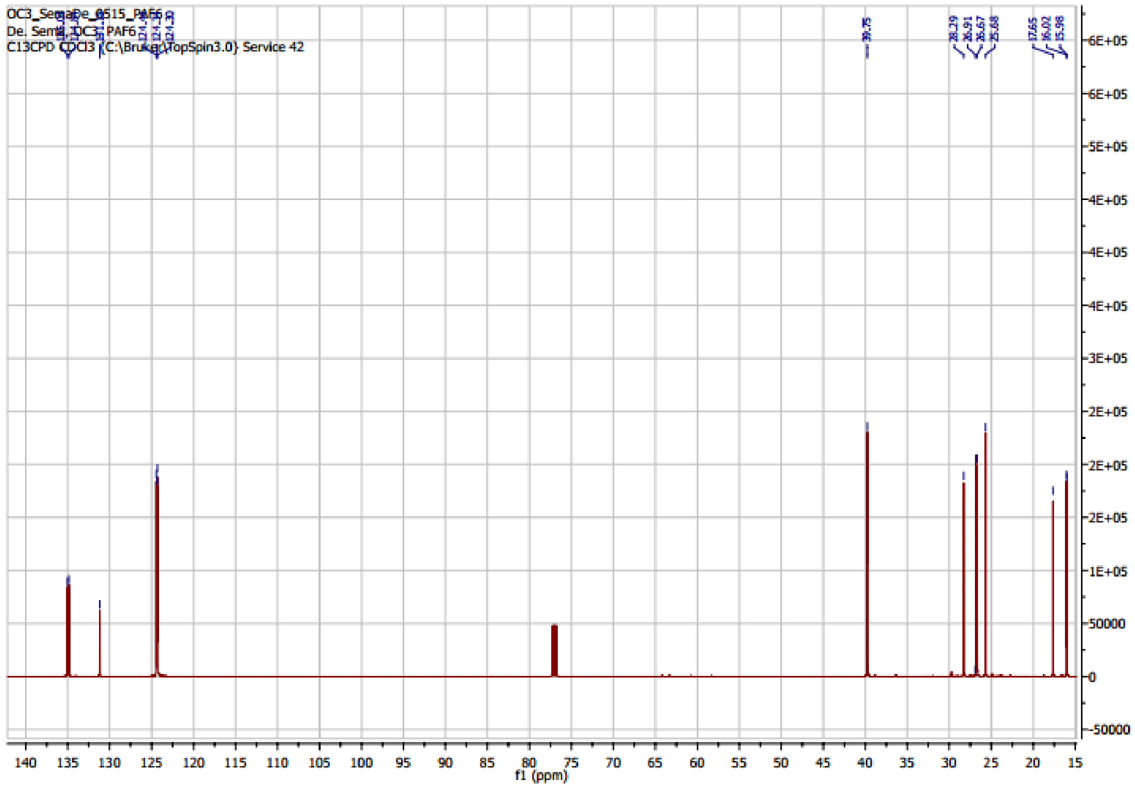

FIGURE S3:^{13}C NMR spectrum (125 MHz, $CDCl_3$) of compound **1**

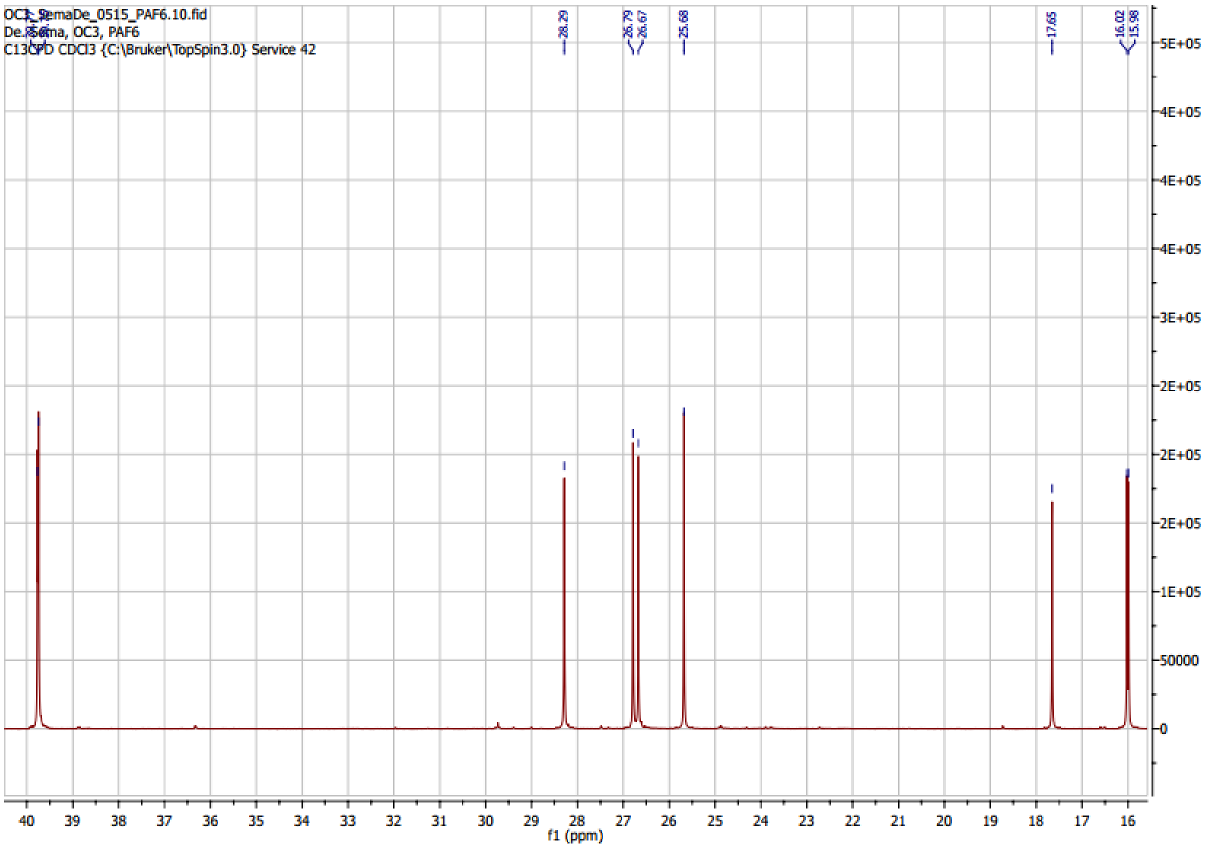

FIGURE S3a: ^{13}C NMR spectrum (125 MHz, $CDCl_3$) of compound **1** (extended)

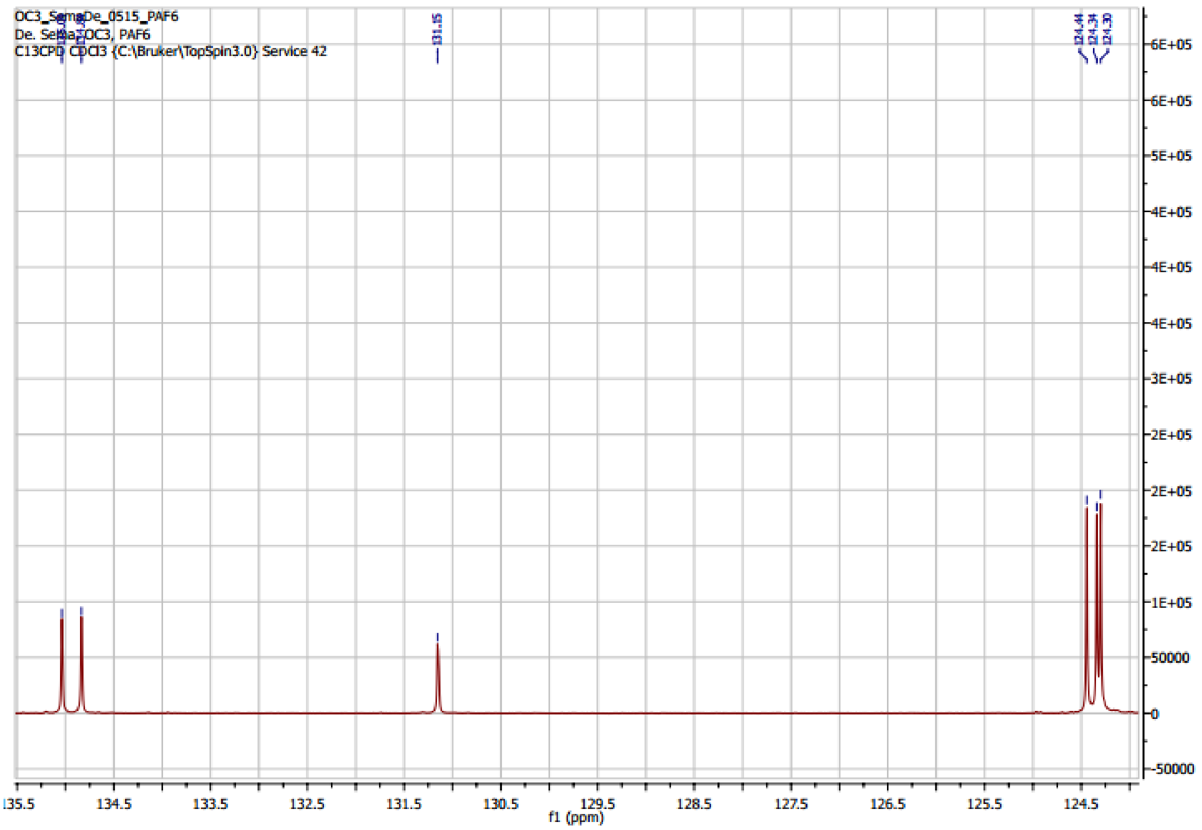

FIGURE S3b: ^{13}C NMR spectrum (125 MHz, $CDCl_3$) of compound **1** (extended)

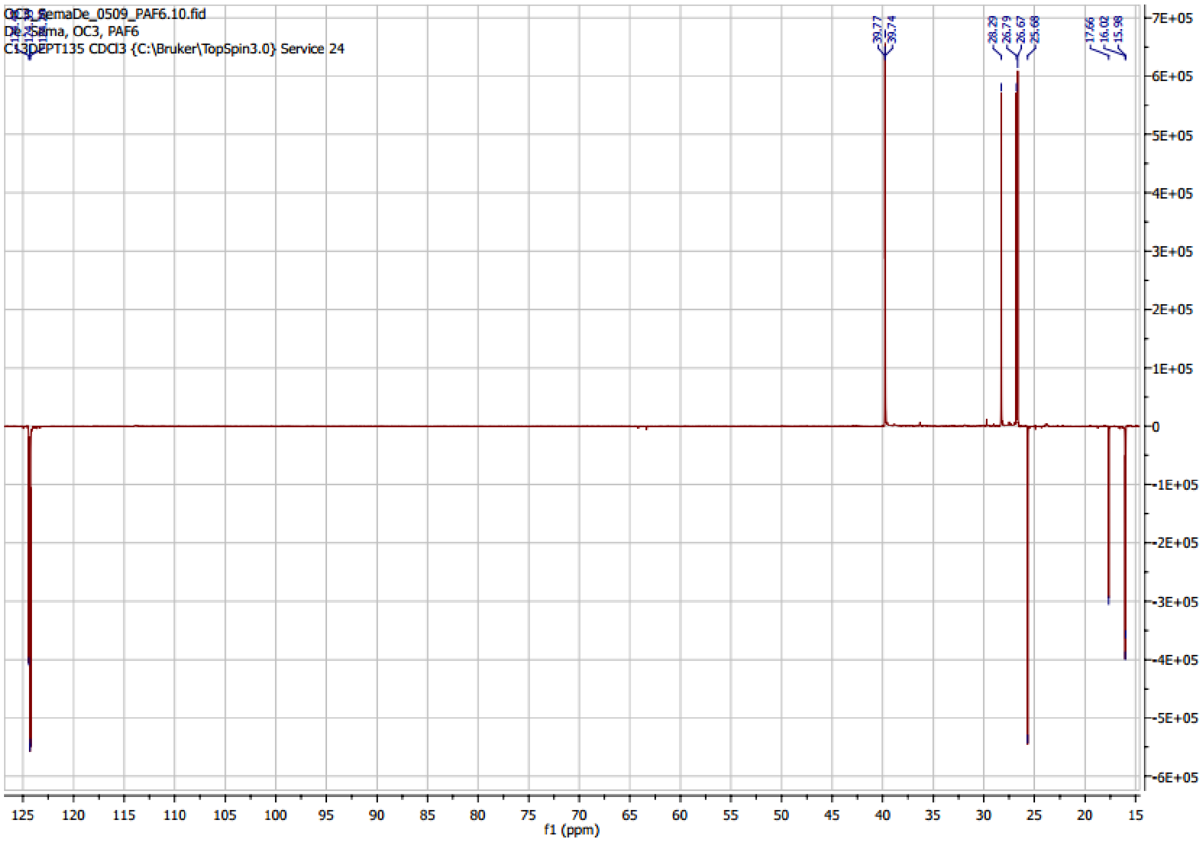

FIGURE S4: DEPT-135 NMR spectrum (125 MHz, $CDCl_3$) of compound **1**

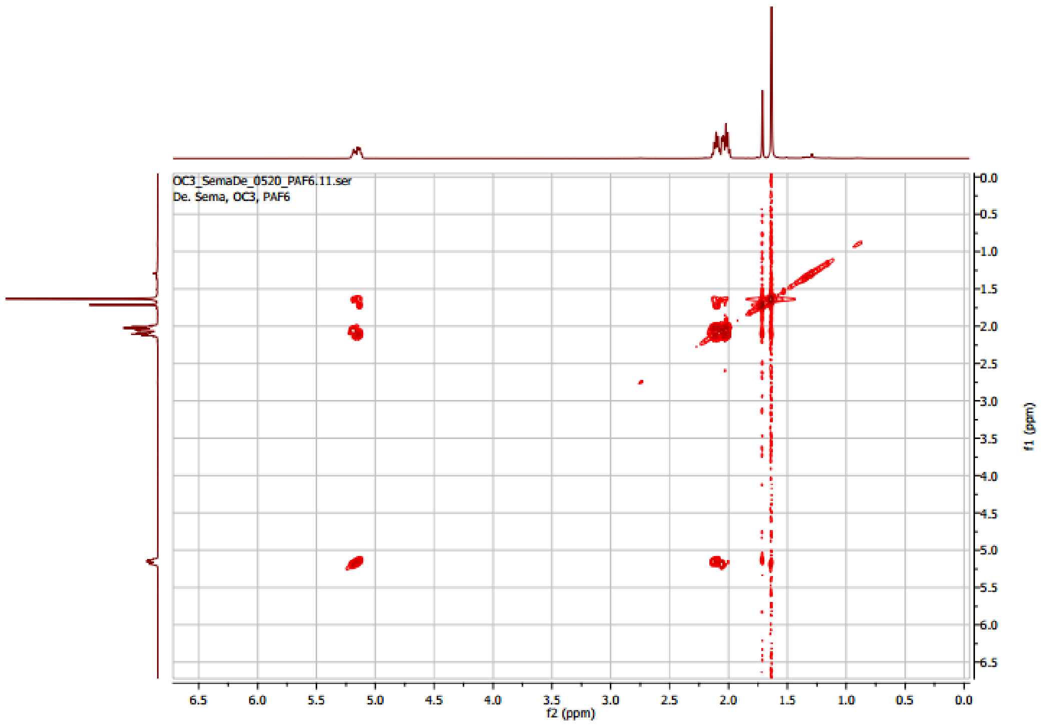

FIGURE S5: COSY-45 spectrum of compound **1**

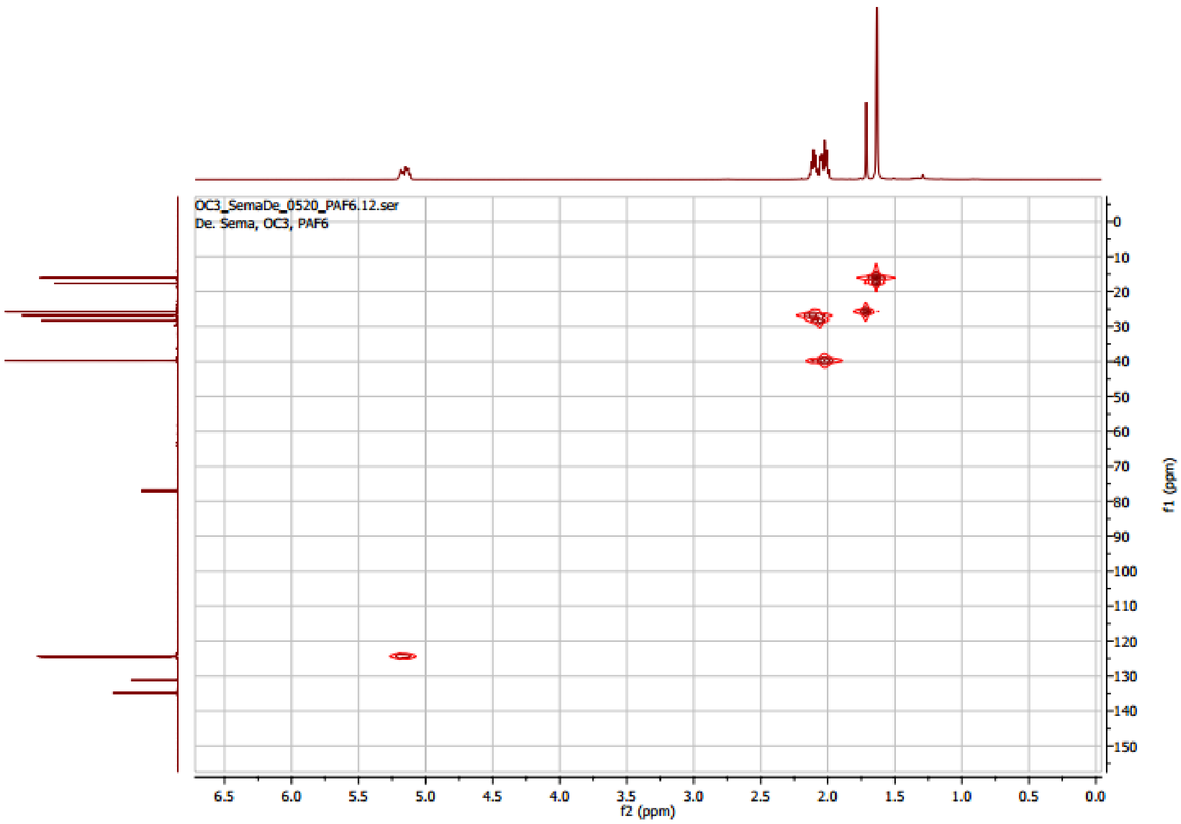

FIGURE S6: HMQC spectrum of compound **1**

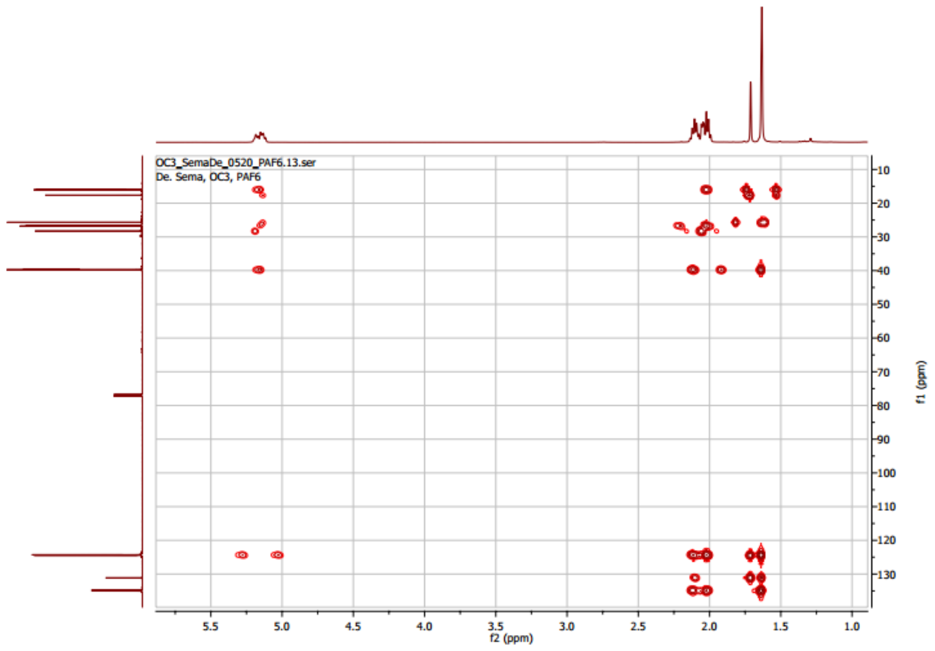

FIGURE S7: HMBC spectra of compound 1

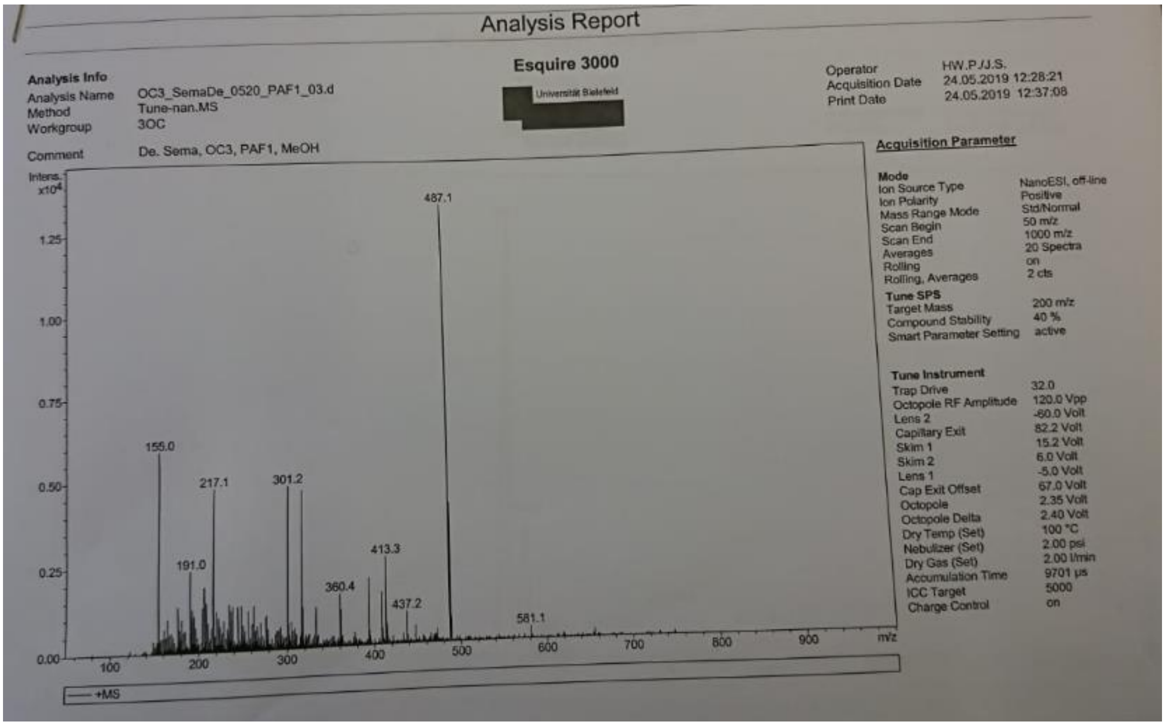

FIGURE S8: Mass spectrum ESI of compounds **2**

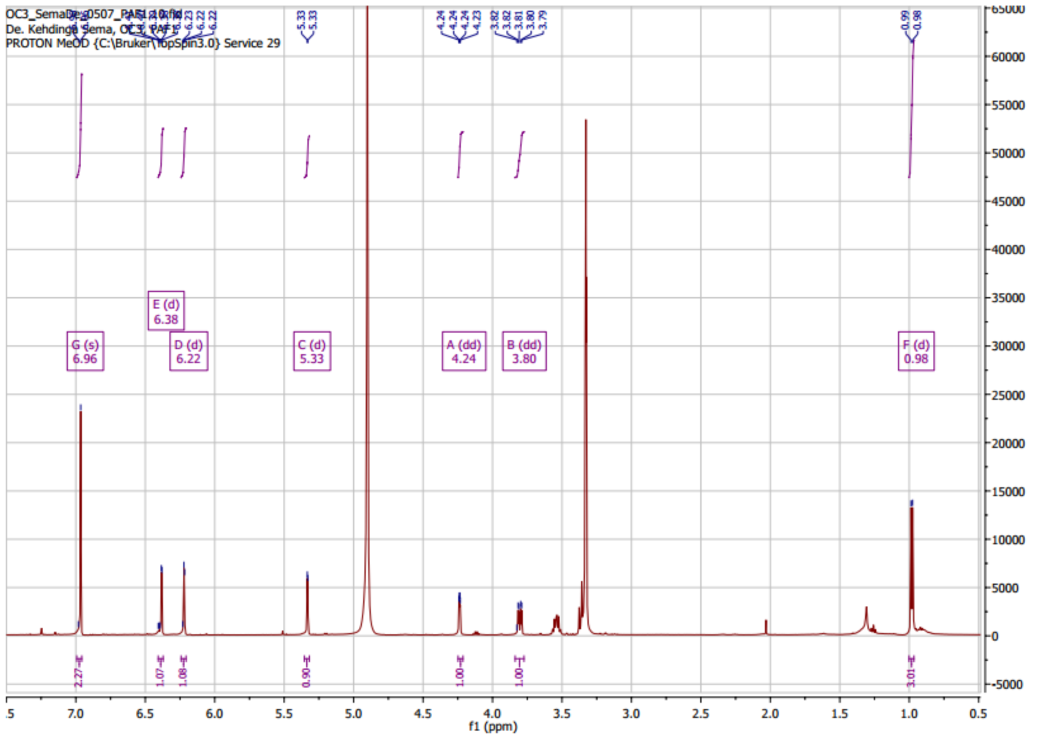

FIGURE S9: ^{1}H NMR spectrum (500 MHz, CD_3OD) of compound **2**

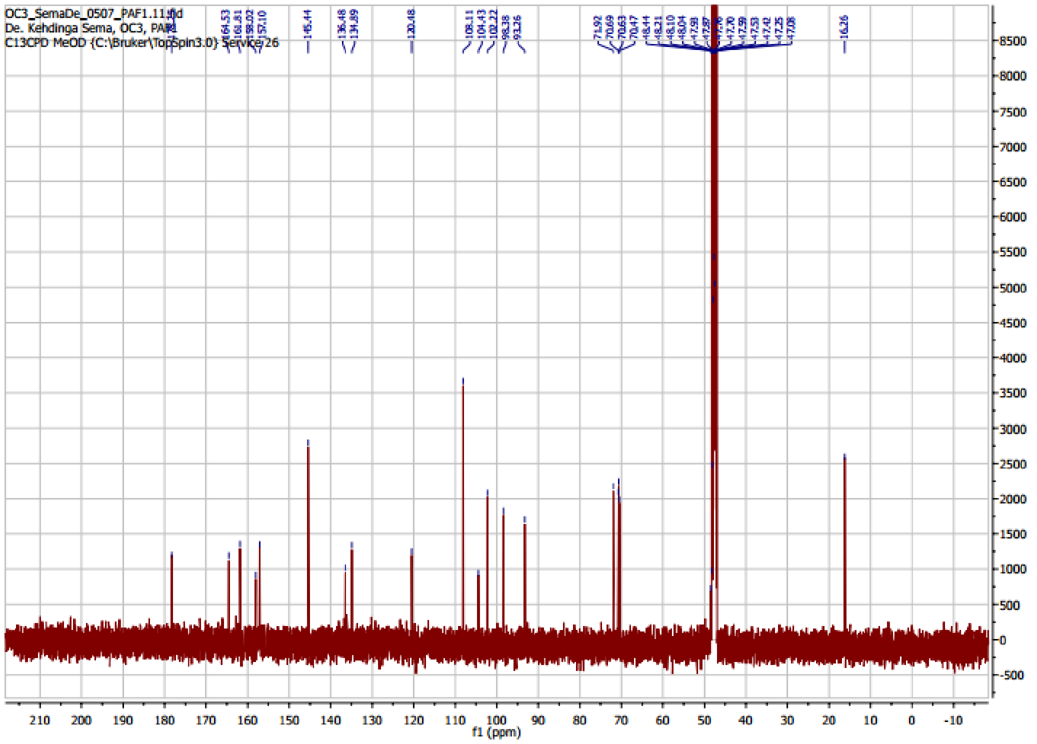

FIGURE S10: ^{13}C NMR spectrum (125 MHz, CD_3OD) of compound **2**

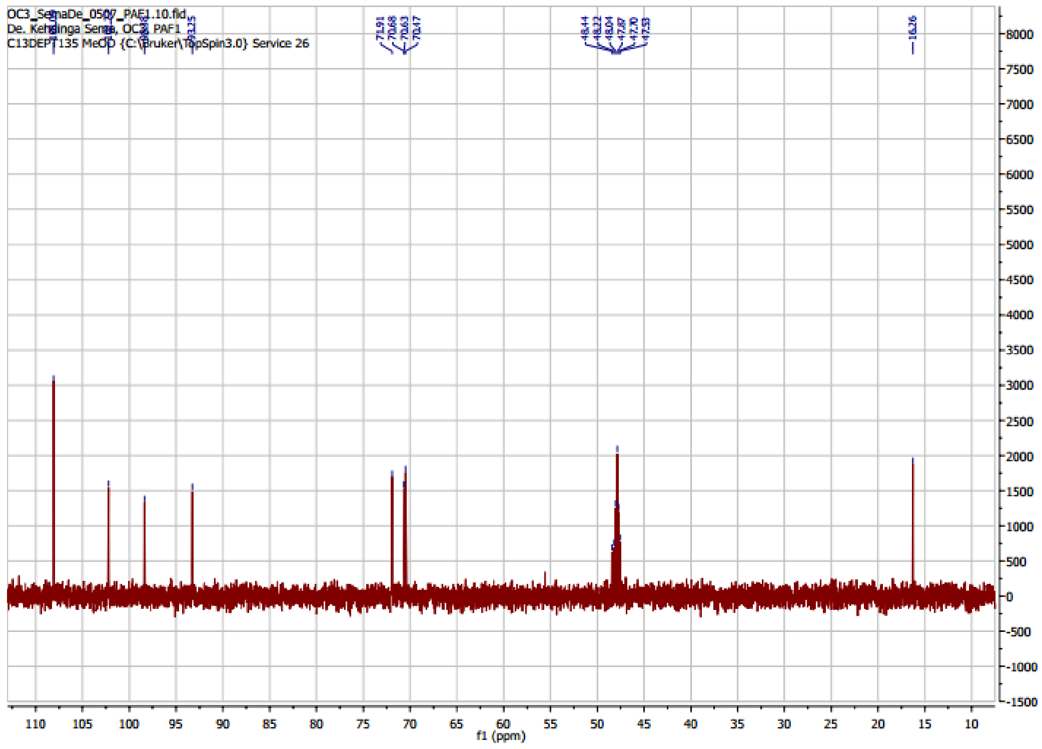

FIGURE S11: DEPT-135 spectrum (125 MHz, CD_3OD) of compound **2**

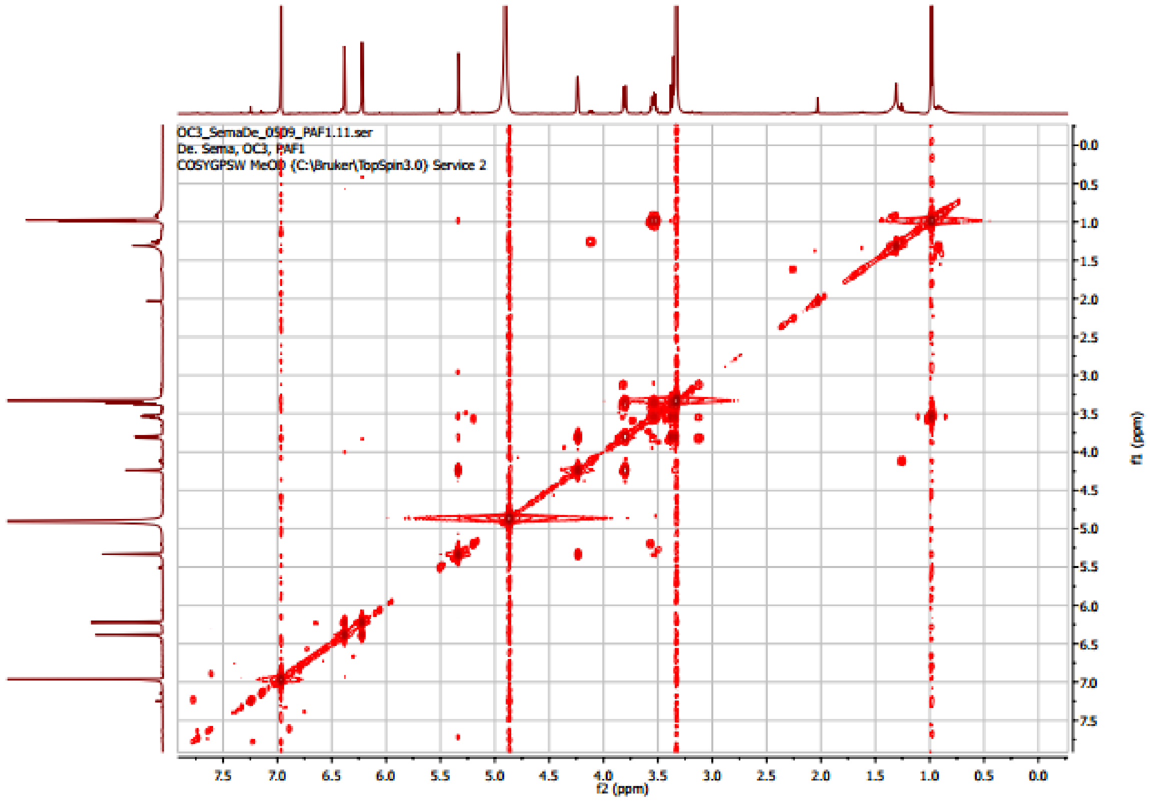

FIGURE S12**:** COSY-45 spectrum of compound **2**

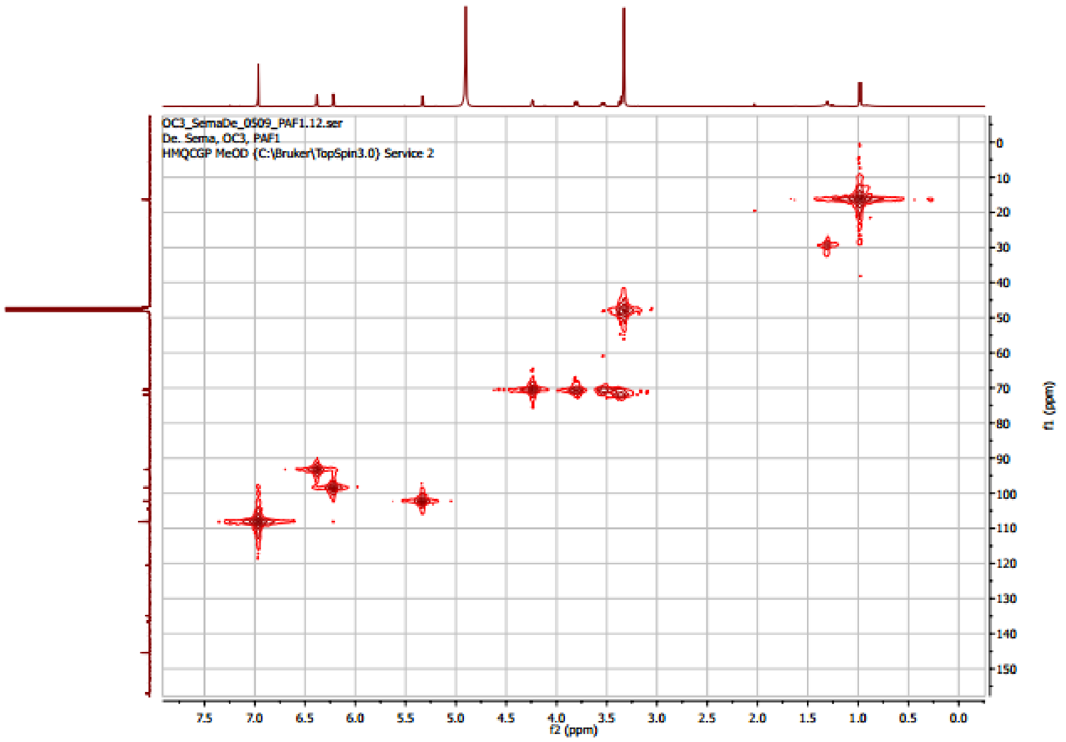

FIGURE S13**:** HMQC spectrum of compound **2**

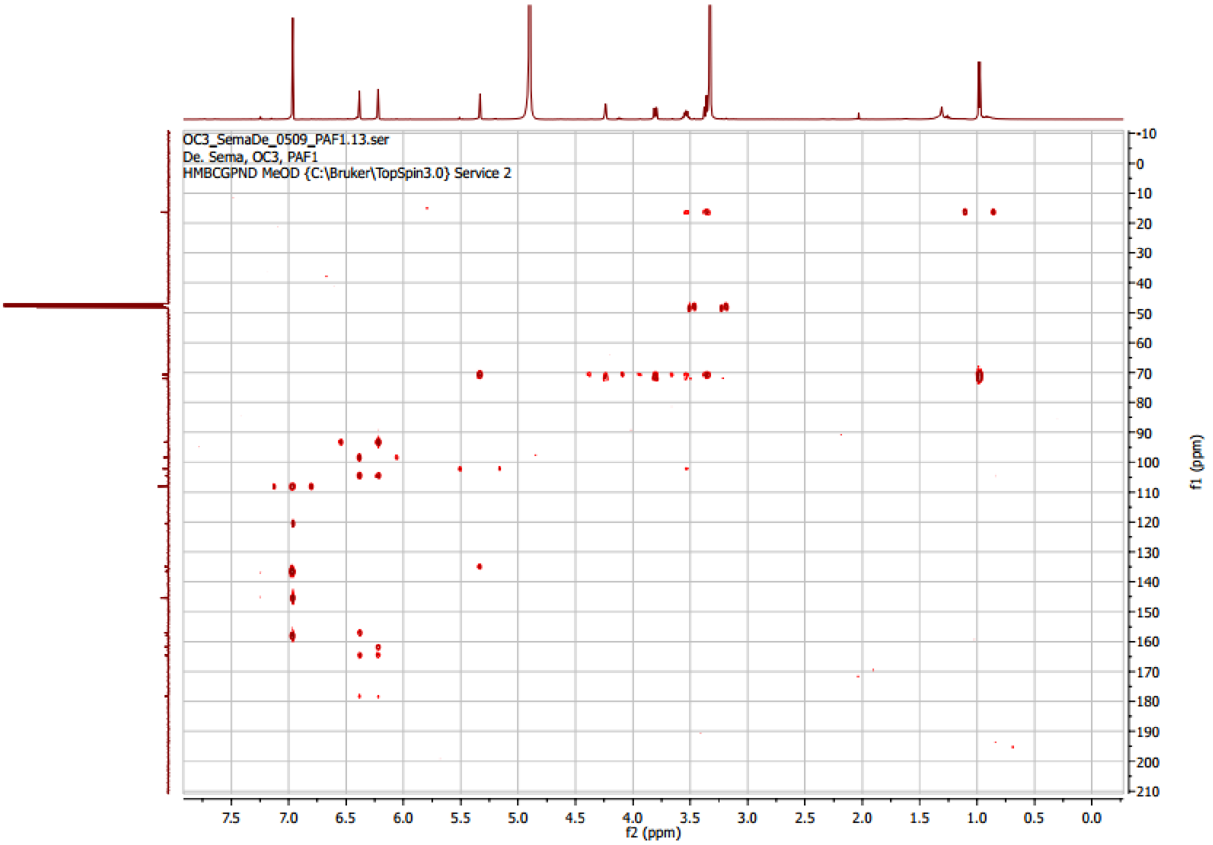

FIGURE S14: HMBC spectrum of compound **2**

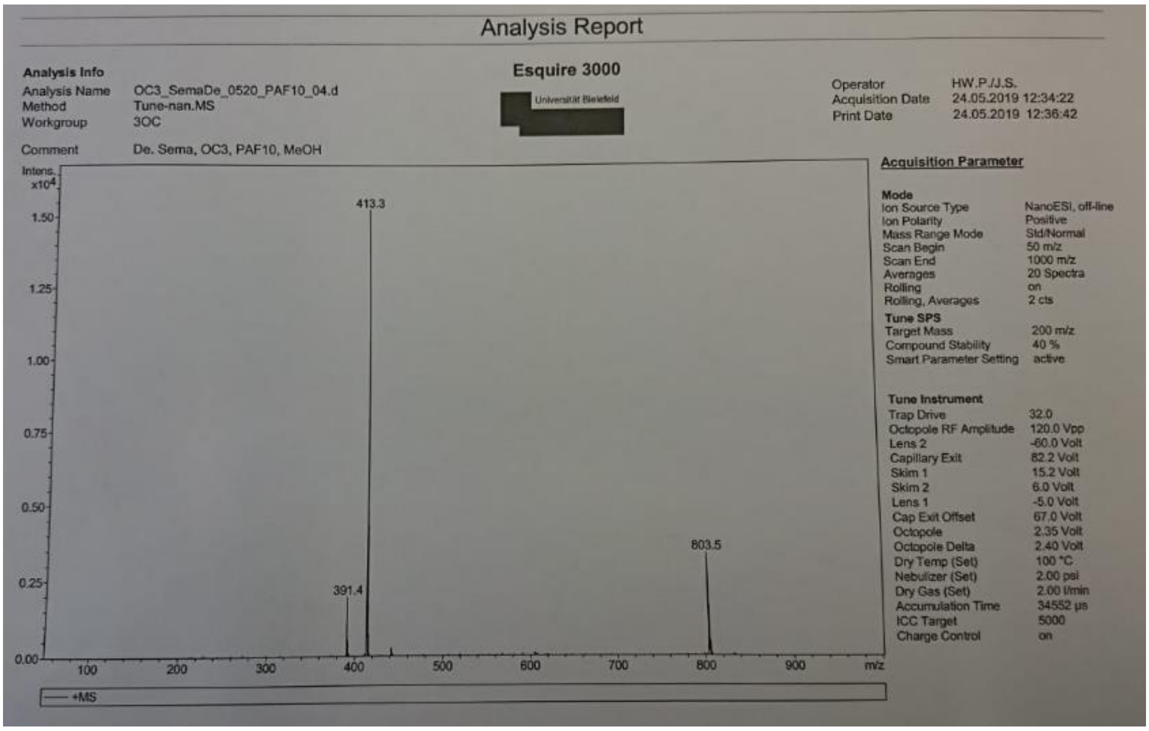

FIGURE S15: Mass spectrum ESI(+) of compound 3

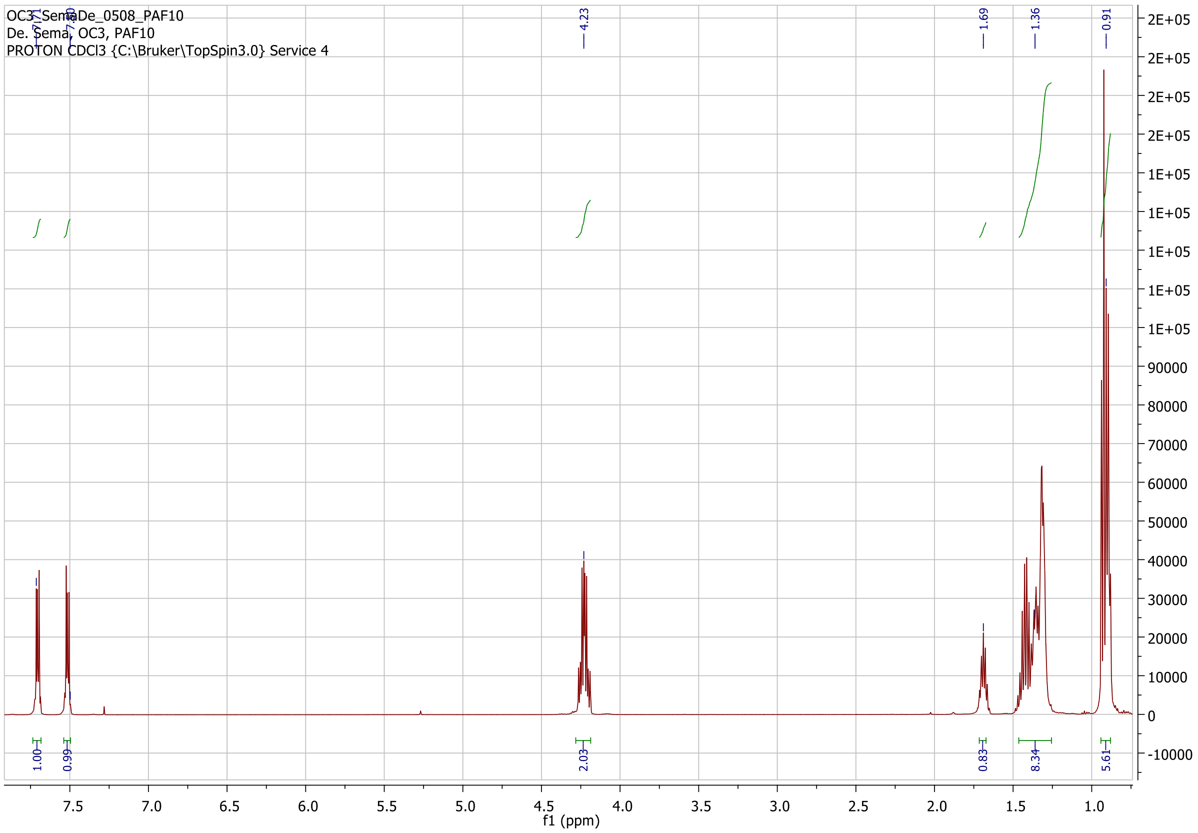

FIGURE S16: ^{1}H NMR spectrum (500 MHz, $CDCl_3$) of compound **3**

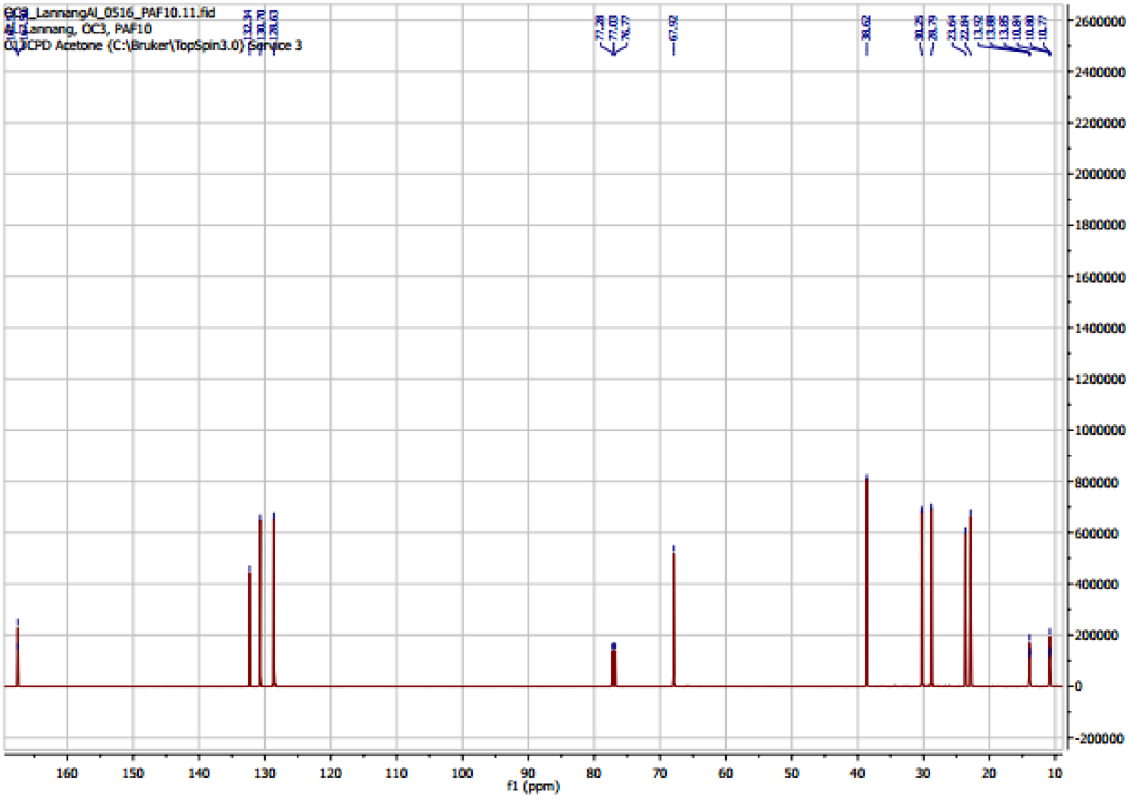

FIGURE S17 : ^{13}C NMR spectrum(125 MHz, $CDCl_3$) of compound **3**

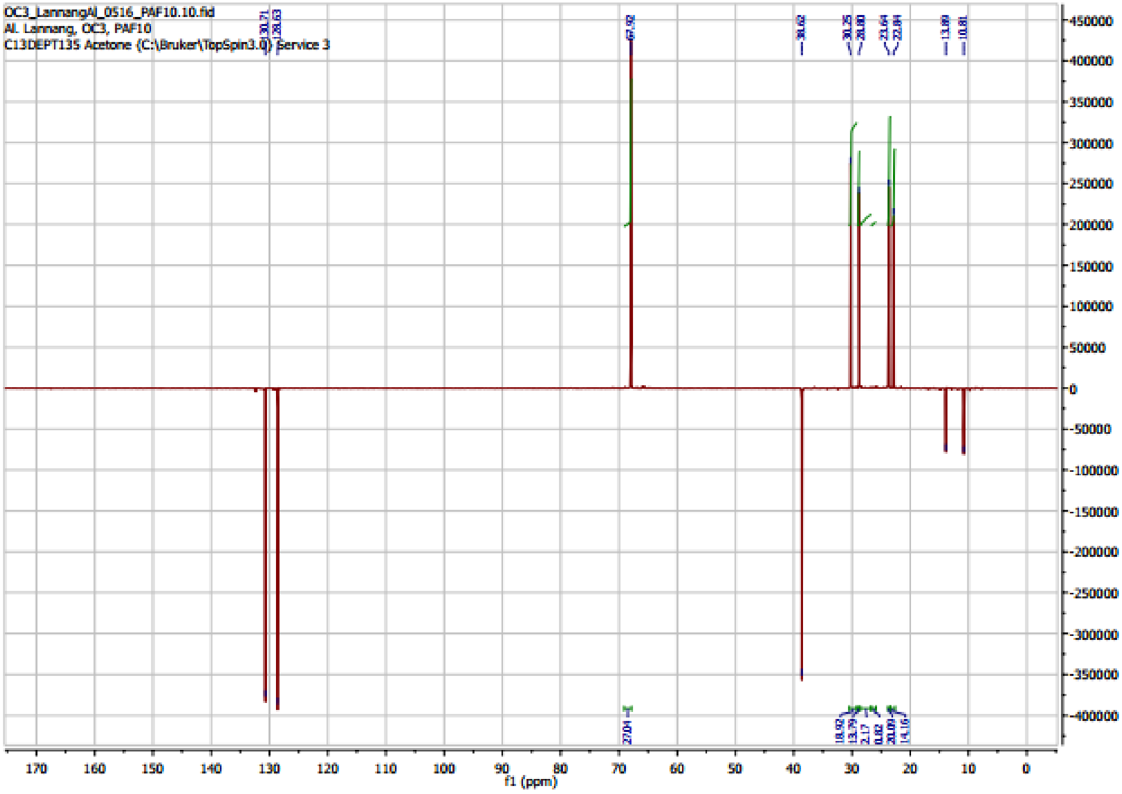

FIGURE S18: DEPT-135 NMR spectrum (125 MHz, $CDCl_3$) of compound **3**

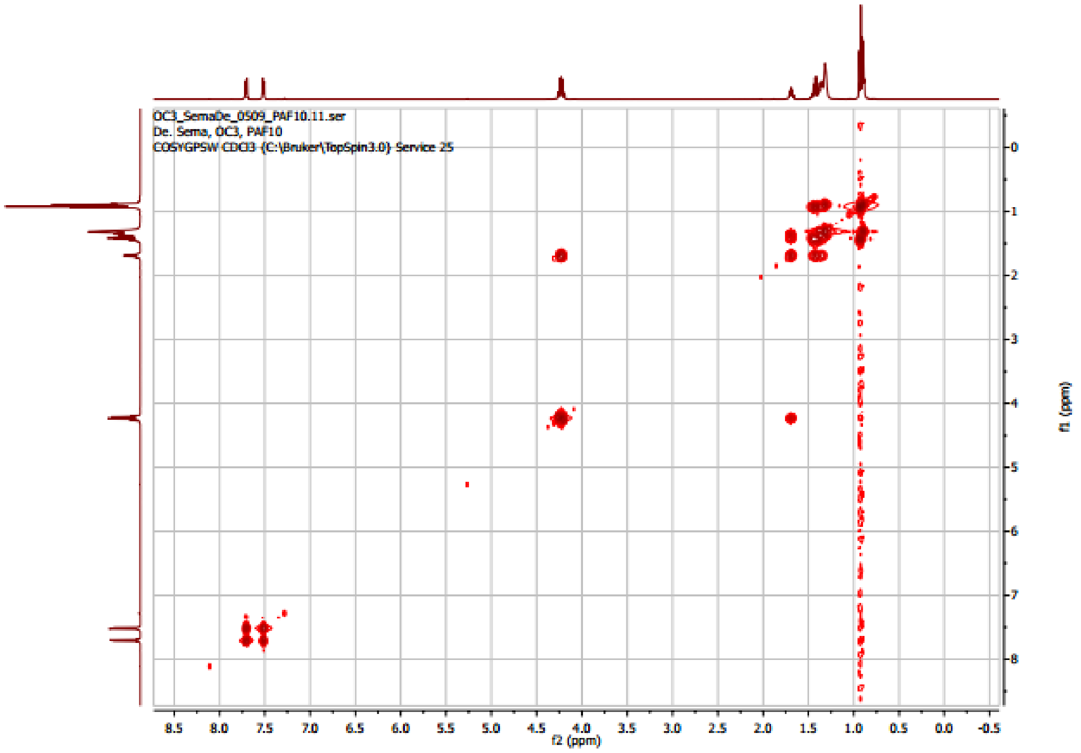

FIGURE S19: COSY-45 spectrum of compound **3**

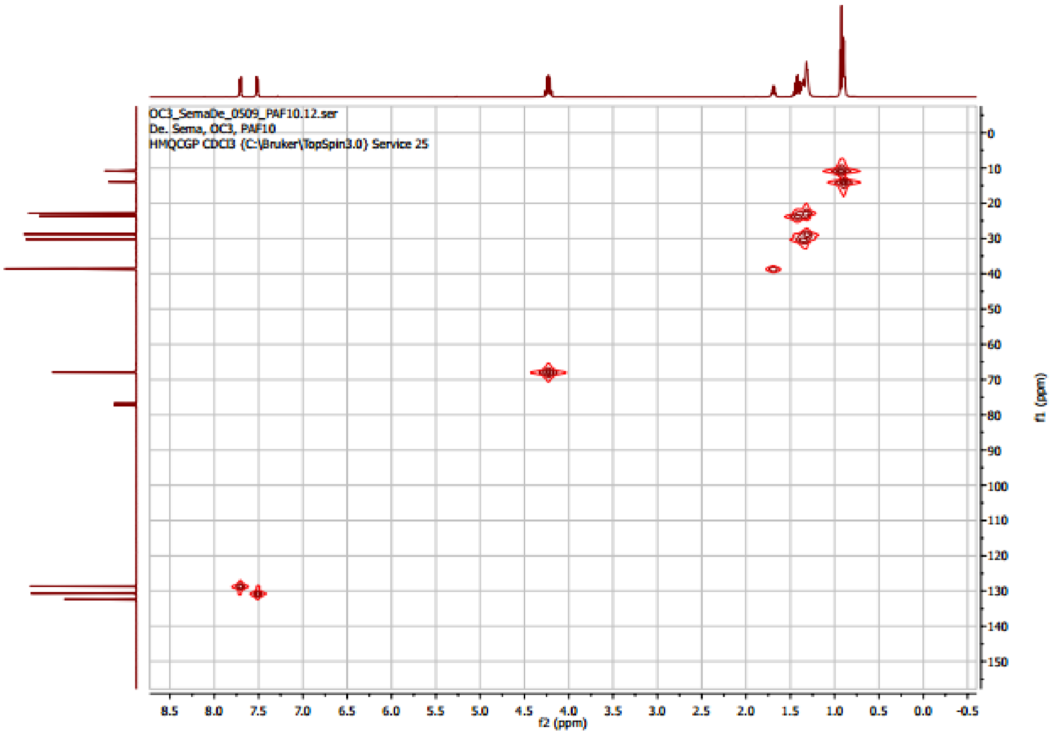

FIGURE S20**:** HMQC spectrum of compound **3**

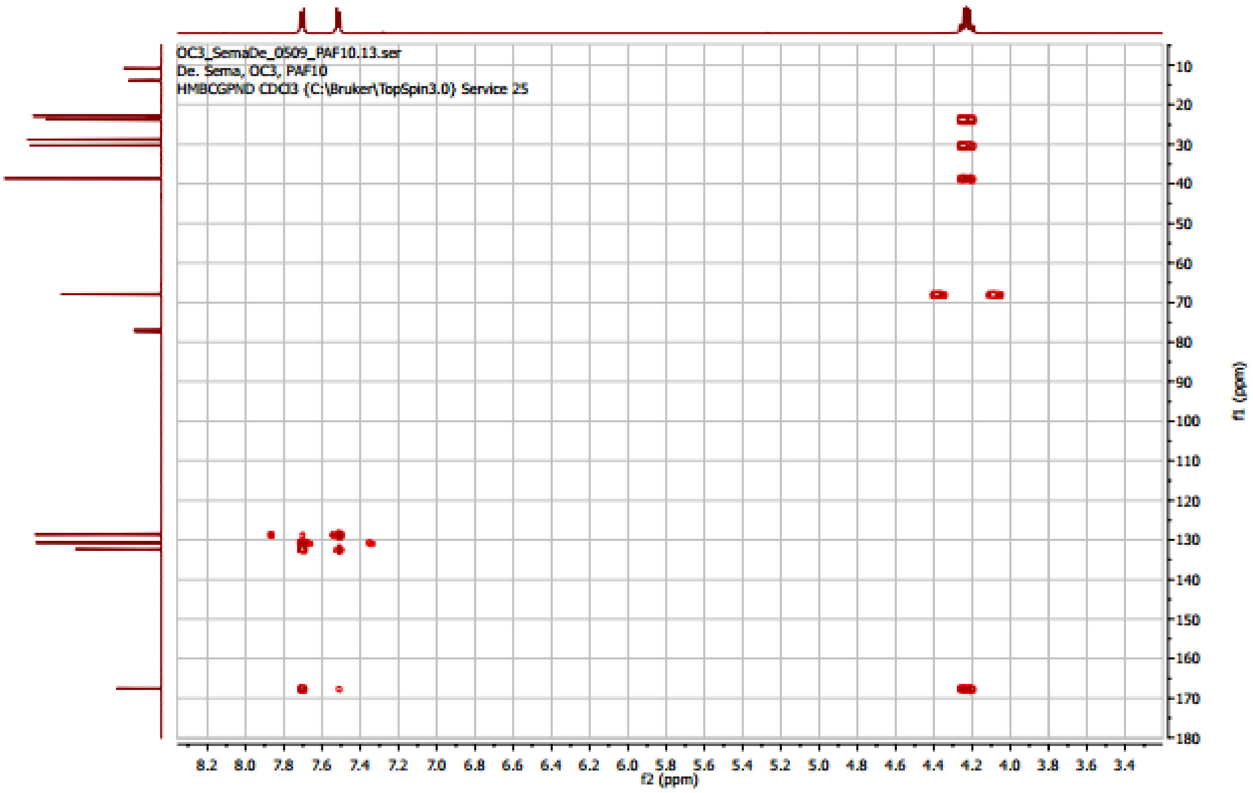

FIGURE S21: HMBC spectrum of compound **3**

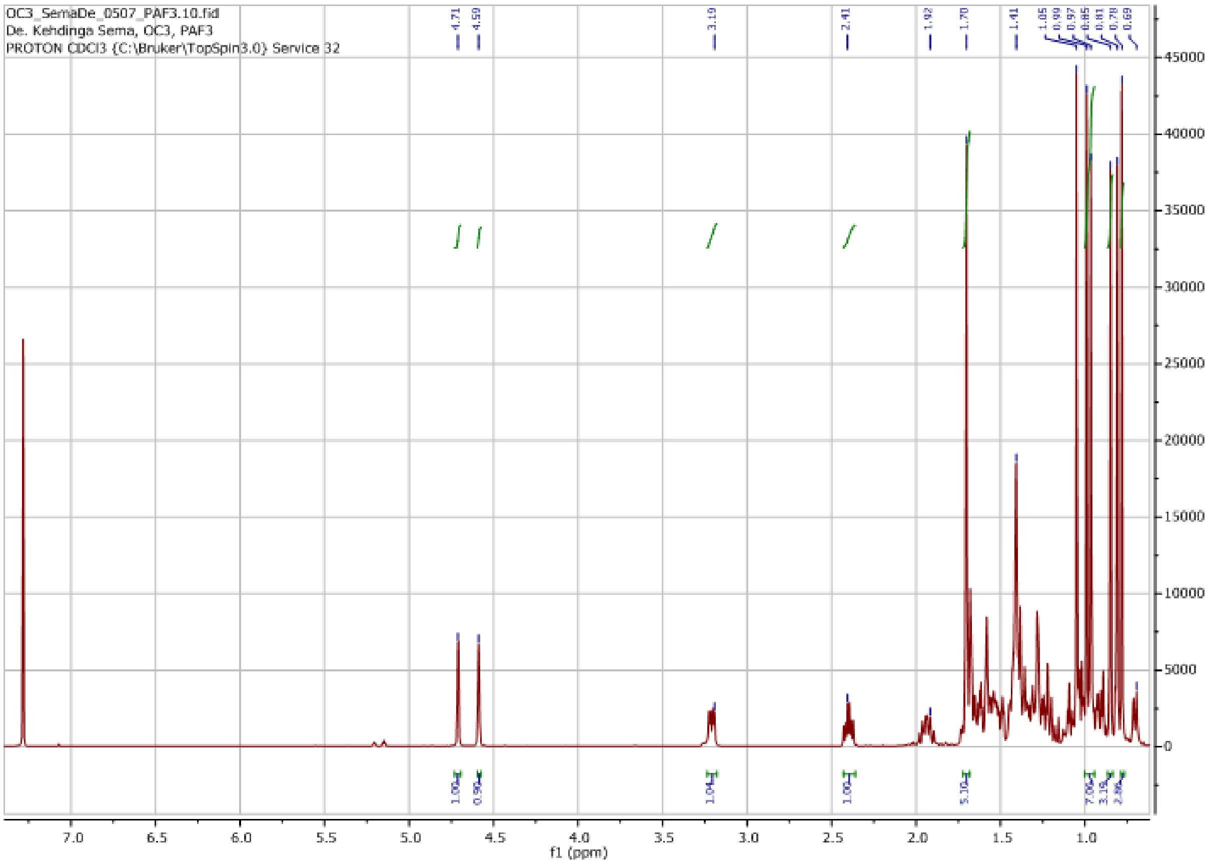

FIGURE S22: ^{1}H NMR spectrum (500 MHz, $CDCl_3$) of compound **5**

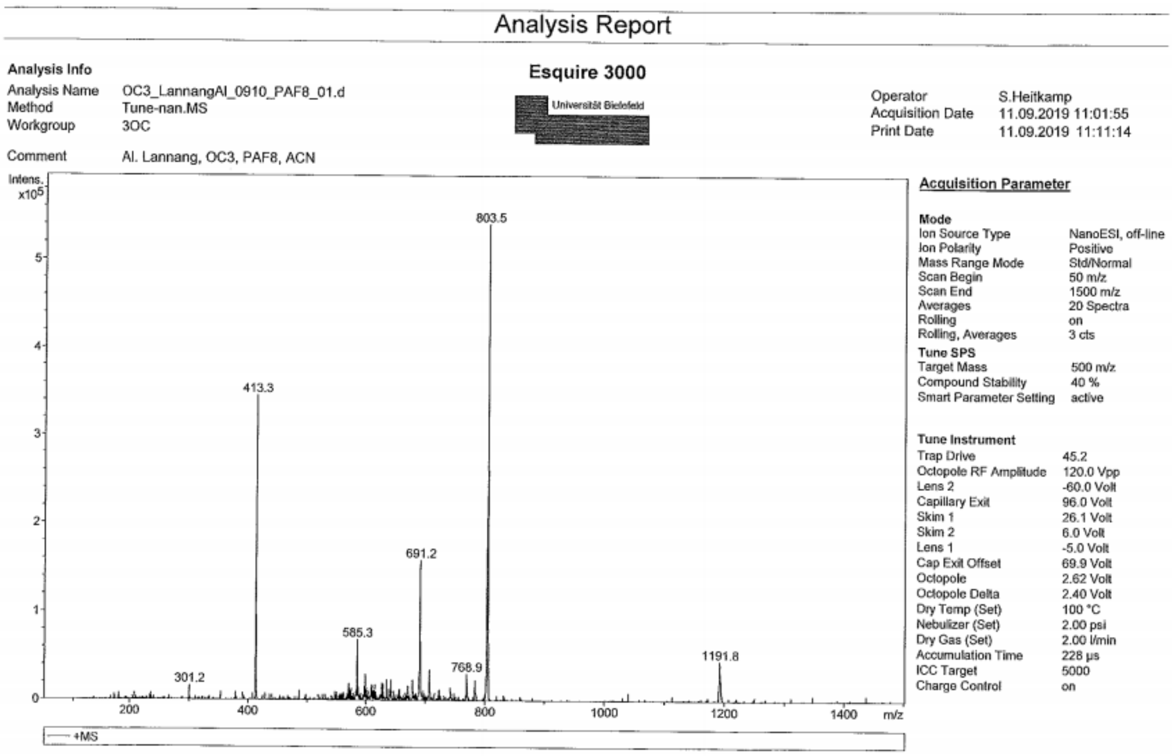

FIGURE S23: Mass spectrum ESI (+) of compound **7**

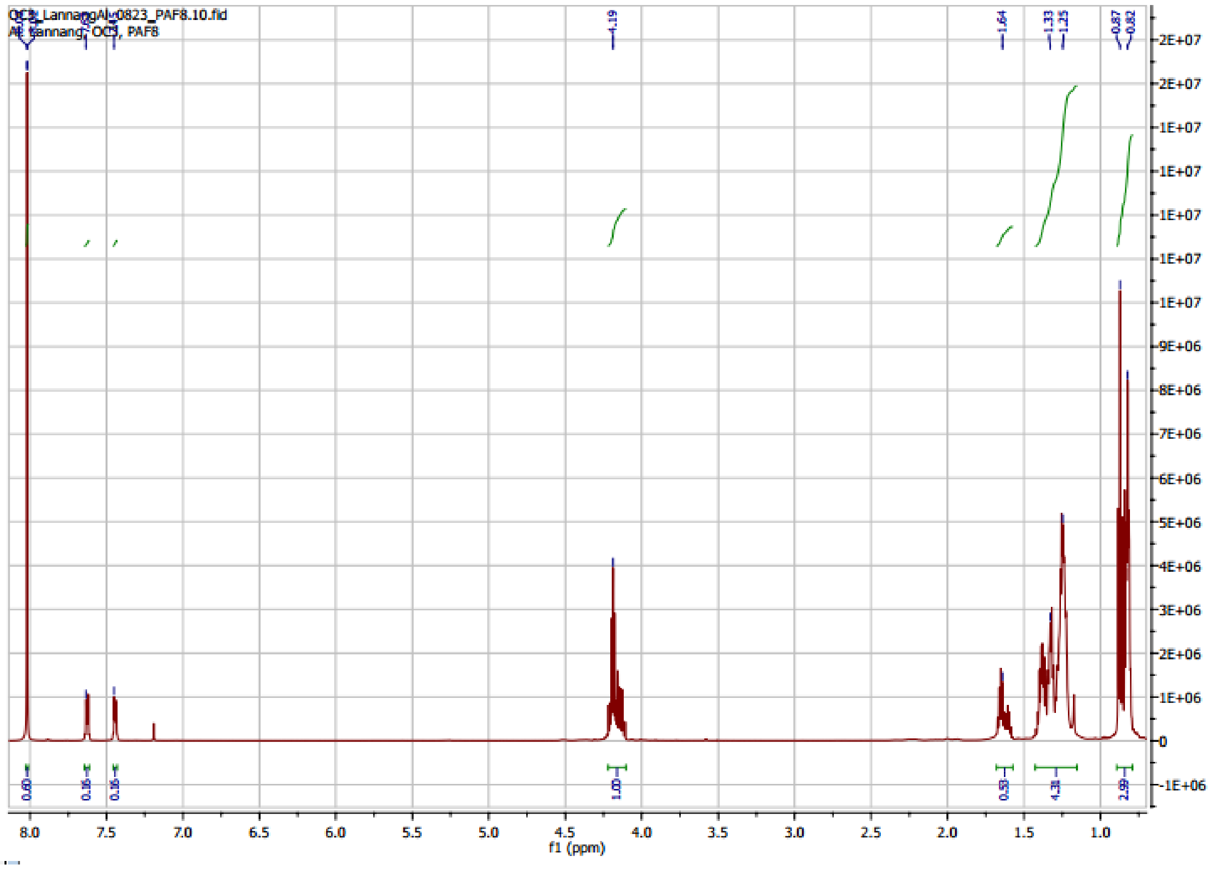

FIGURE S24: ^{1}H NMR (500 MHz, $CDCl_3$) of compound **7**

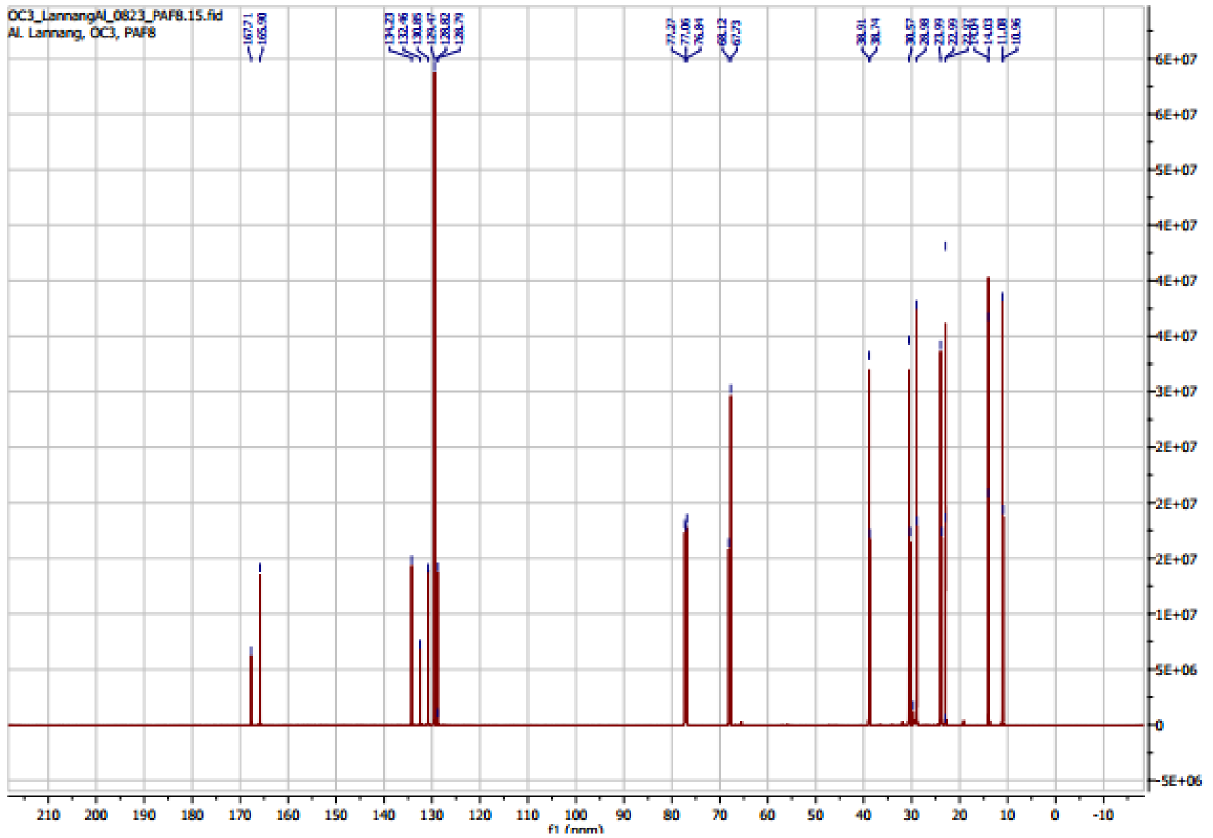

FIGURE S25: ^{13}C NMR (125 MHz, $CDCl_3$) of compound **7**

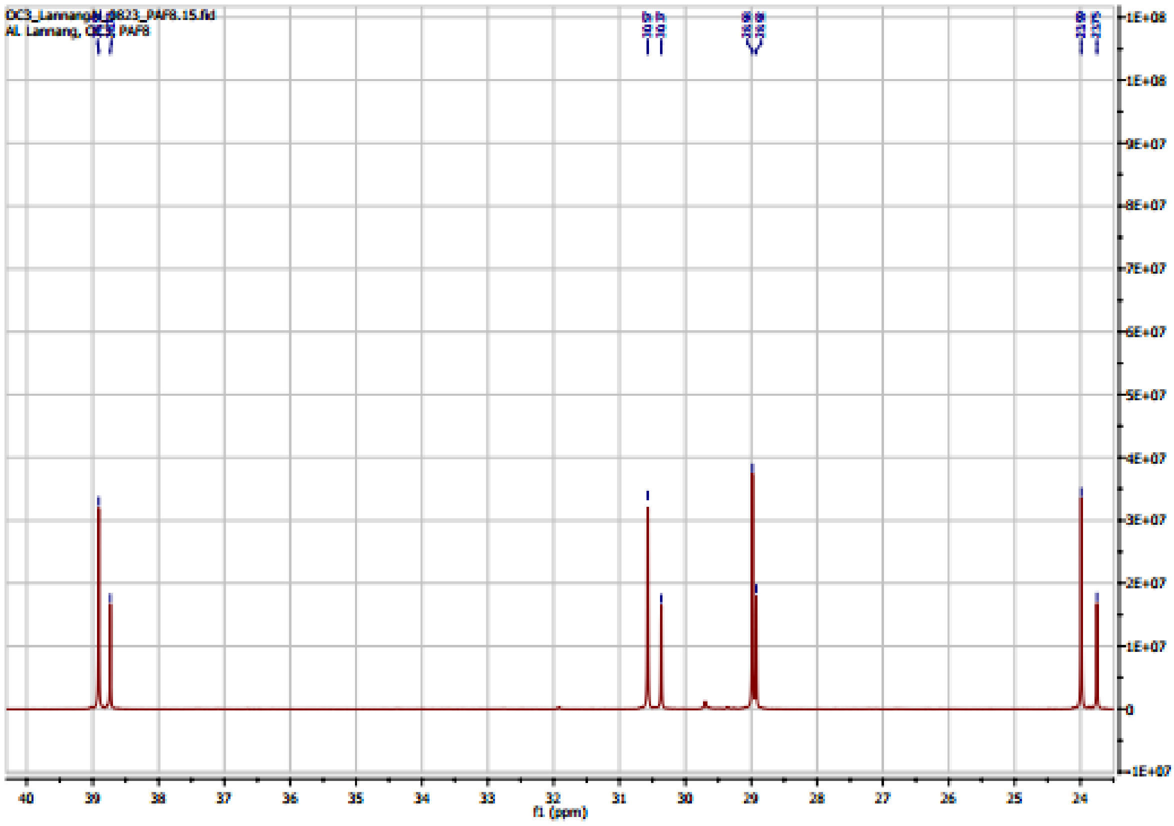

FIGURE S25a: ^{13}C NMR (125 MHz, $CDCl_3$) of compound **7**

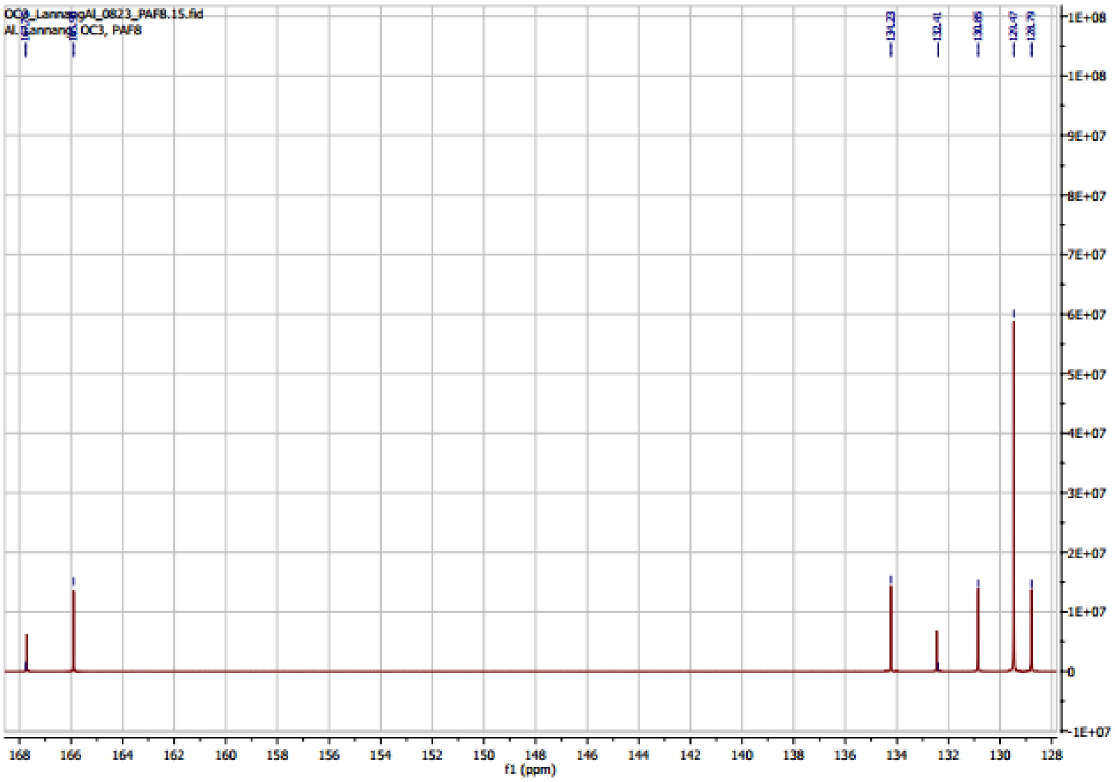

FIGURE S25b: ^{13}C NMR (125 MHz, $CDCl_3$) of compound **7**

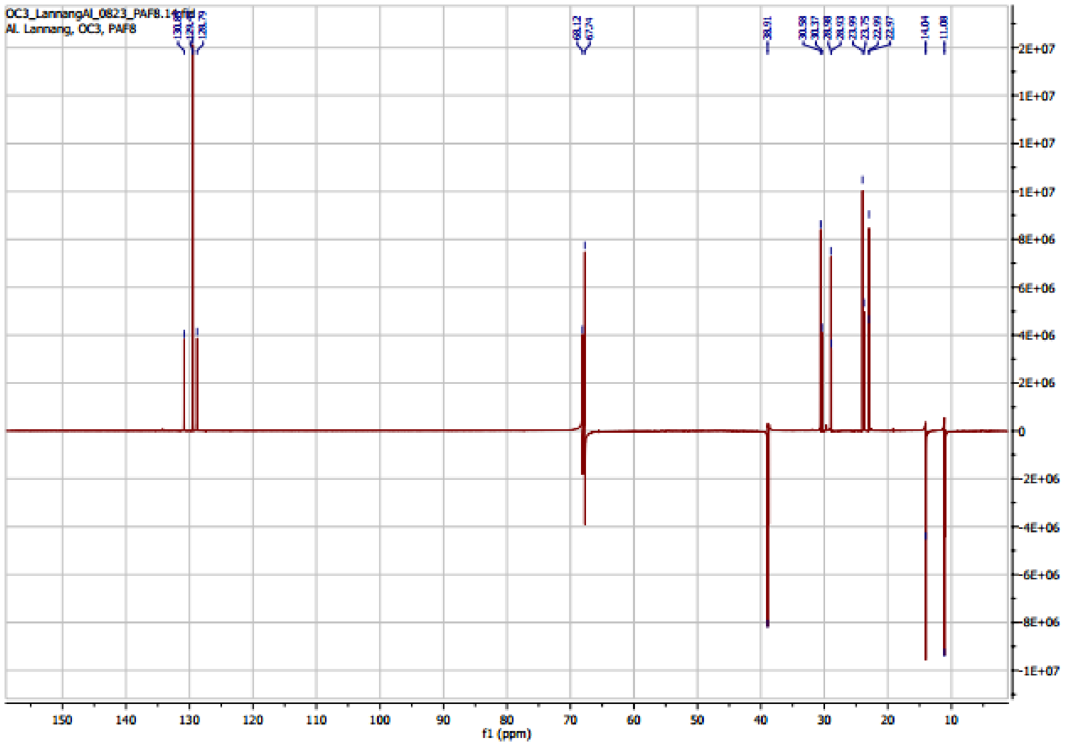

FIGURE S26:^{13}C NMR (125 MHz, $CDCl_3$) of compound **7**

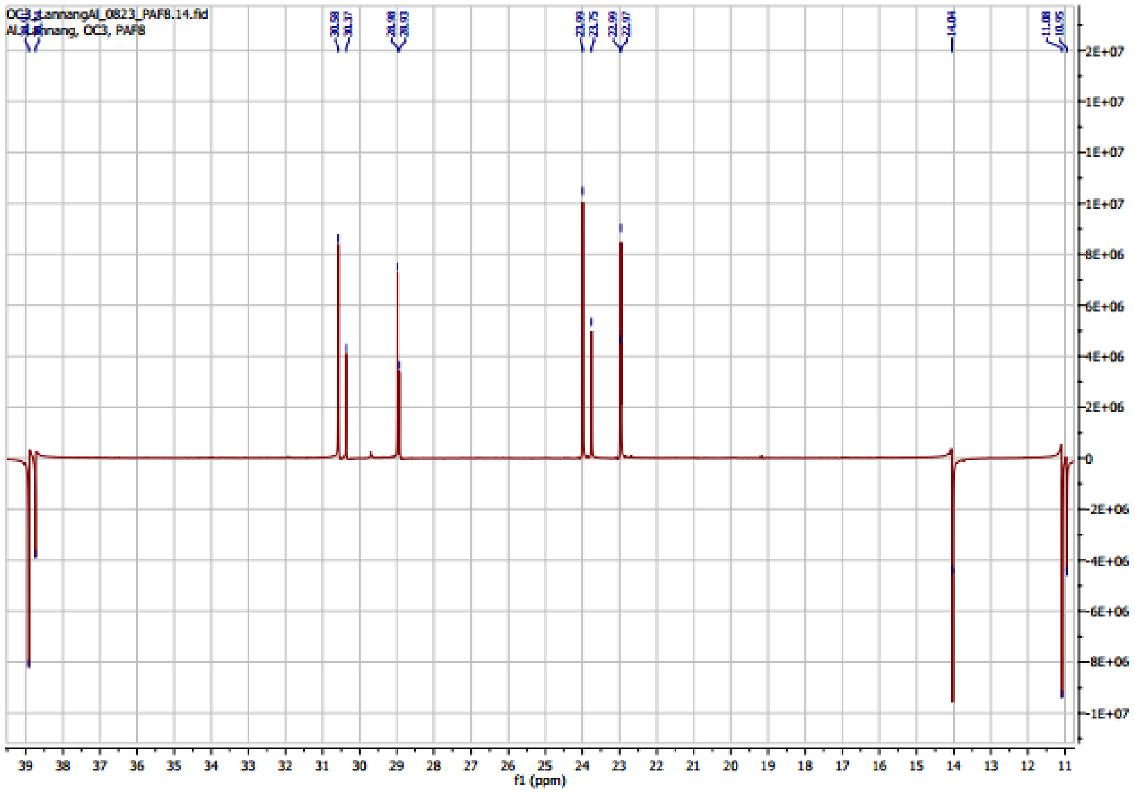

FIGURE S26a:^{13}C NMR (125 MHz, $CDCl_3$) of compound **7**

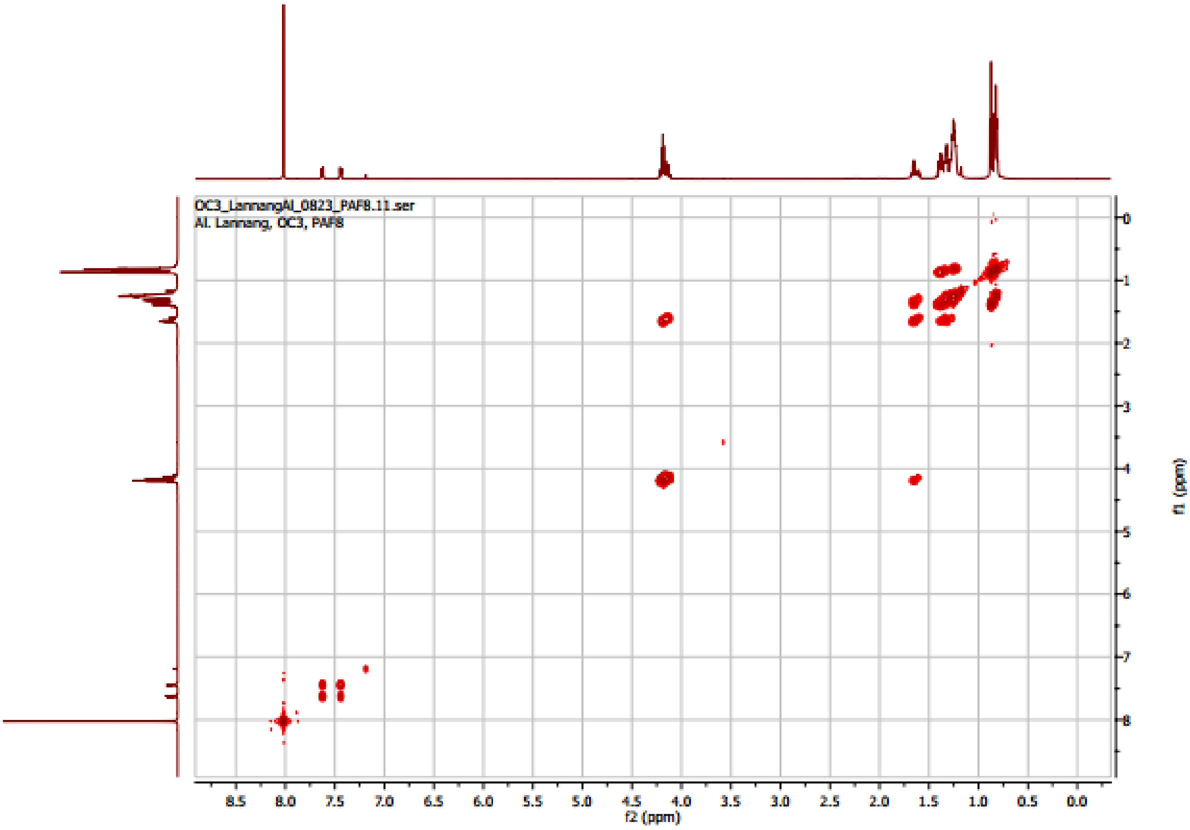

FIGURE S27: COSY-45 spectrum of compound **7**

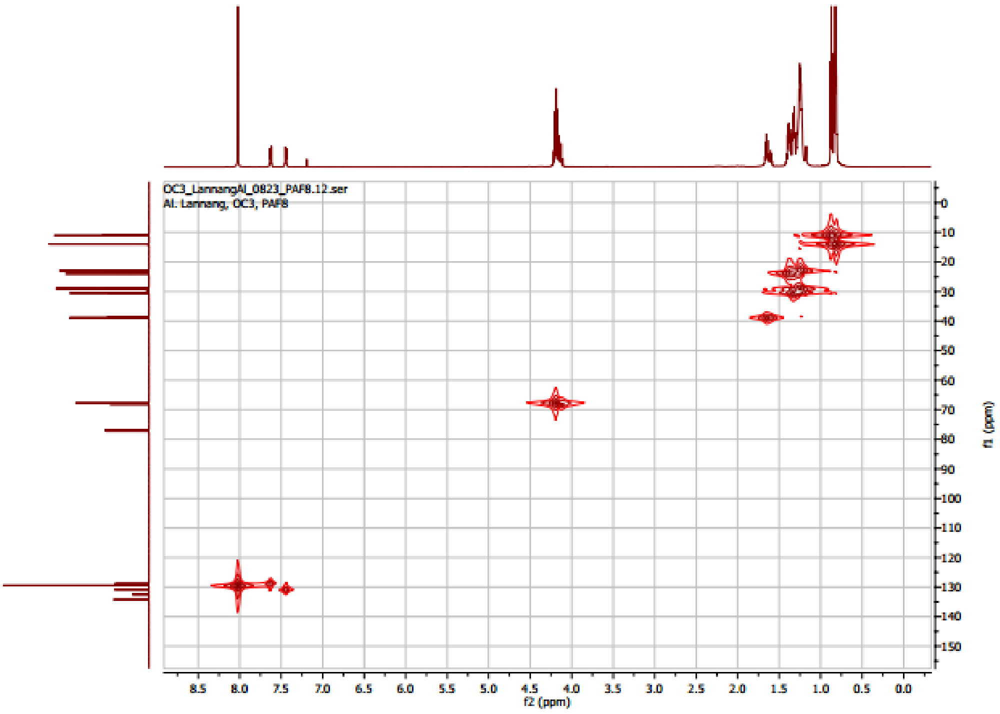

FIGURE S28: HMQC spectrum of compound **7**

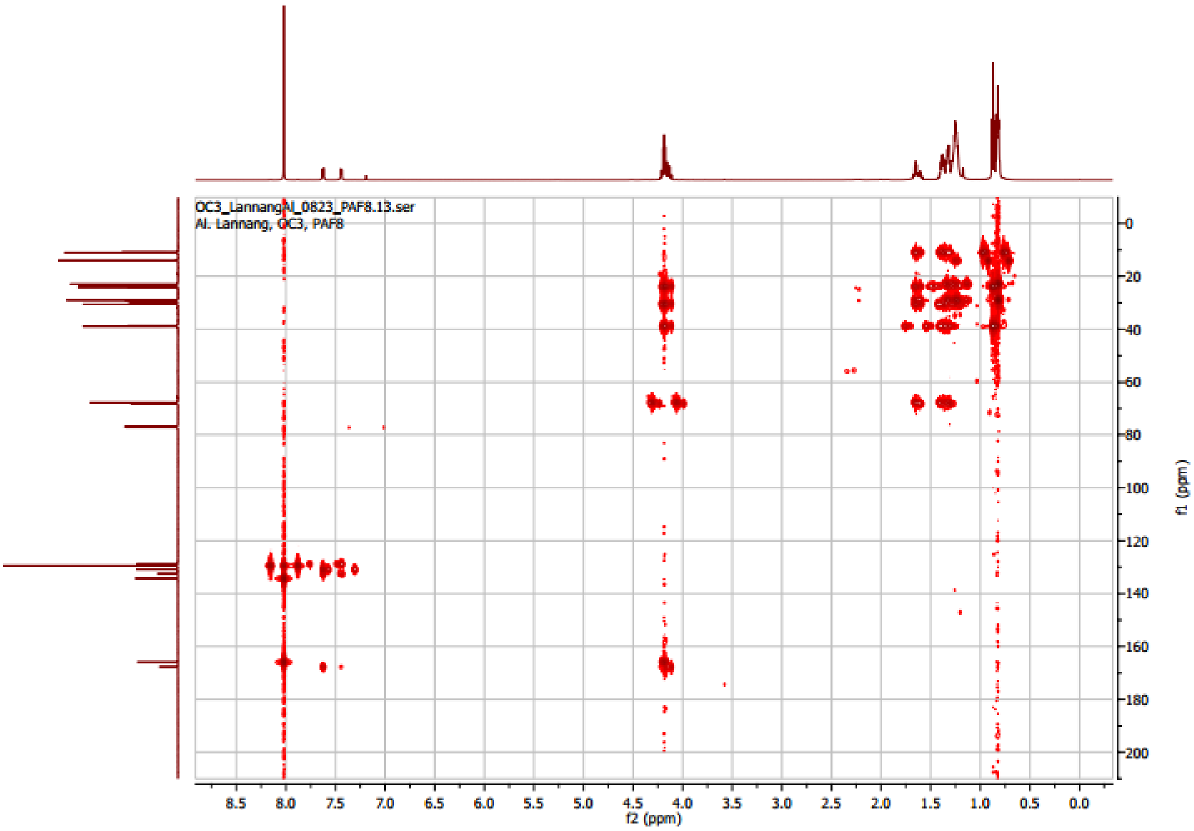

FIGURE S29: HMBC spectrum of compound **7**
